# Social support and mental health in late adolescence are correlated for genetic, as well as environmental, reasons

**DOI:** 10.1038/s41598-017-13449-2

**Published:** 2017-10-12

**Authors:** R. Adele H. Wang, Oliver S. P. Davis, Robyn E. Wootton, Abigail Mottershaw, Claire M. A. Haworth

**Affiliations:** 1 School of Experimental Psychology, University of Bristol, Bristol, BS8 1TU United Kingdom; 2 MRC Integrative Epidemiology Unit, University of Bristol, Bristol, BS8 2BN United Kingdom; 3 School of Social and Community Medicine, University of Bristol, Bristol, BS8 2PS United Kingdom

## Abstract

Late adolescence is a crucial, but underexplored, developmental stage with respect to the aetiology of social support. These individuals are experiencing many major life changes and social support can help them adjust to the associated environmental stressors of this time. Using 1,215 18-year-old twin pairs from the Twins Early Development Study, we collected measures of two indices of support: support quality and support quantity, as well as wellbeing and depression. Both support indices were moderately heritable (55% and 49%, respectively), an interesting finding given the many environmental changes that late adolescents are encountering that could be environmentally altering their social network structures. Finding a genetic influence on support suggests the presence of gene-environment correlation whereby individuals create and perceive their supportive environment based upon their genetic predispositions. Shared genetic influences mediated the moderate phenotypic correlation (mean r = 0.46) between support and mental health. Genetic correlations were higher between support quality and mental health (mean rA = 0.75), than between support quantity and mental health (mean rA = 0.54), reflecting the phenotypic pattern. This suggests that interventions should focus more on making late adolescents aware of the support quality around them than encouraging them to increase their social network size.

## Introduction

Adolescence is an important period of biological, cognitive and emotional development. Wellbeing levels tend to decrease during this time^1^. Many mental health problems also tend to emerge^2,3^ and can be predictive of occurrence or recurrence of mental illness in later adulthood^4^. Late adolescence is an especially interesting period of development as individuals encounter many environmental changes such as moving away from home, transitioning from education to employment, and starting a family, which can provide additional environmental stressors. Social support is a key factor in helping young adults navigate these major life changes^5,6^, but these environmental changes can bring about instability and changes to an individual’s social circle itself, and an individual’s perceptions of support from others can change dramatically during this time^7–9^.

Genetically informative study designs can allow us to investigate the origins of individual differences in social support, providing us with a more in depth understanding of support during this environmentally unstable developmental stage. Our study seeks to understand the aetiology of two dimensions of support: support quality and support quantity. We wish to investigate the phenotypic correlation of these two dimensions with mental health at this age. Furthermore, we wish to expand the sparse literature by exploring the genetic influences that mediate these phenotypic associations using multivariate twin analyses.

Social support has a variety of dimensions^10,11^. A range of operationalisations encompass a mix of factors, such as degree of social integration, function of the social relationship and type of support^12–14^. One notable overlap across many of these definitions is the distinction between a functional definition of support (i.e. the quality of support provided or perceived) and a structural definition (i.e. the network size and quantity of available support). With regards to mental health, quality of support has been shown to be more important than quantity in both adults^15^ and children^16,17^. However, no such distinction has been investigated specifically in late adolescence. This distinction is especially important to look at during this time given the individual’s changes in both social network structure (support quantity)^8^ and support perception (quality)^9^ during this time.

Genetically informative study designs allow us to investigate the reasons why we find individual differences in experiences and outcomes, as well as understand genetic and environmental overlap between these^18^. Social support is typically thought of as a measure of the environment. While environments are assumed to be independent of the individual, studies have repeatedly shown that many environmental measures have a heritable component, supporting a bidirectional model of human-environment inter-relationship^19^. Looking across a range of environments (such as life events, parenting, social support, and marital quality), Kendler and Baker^20^ found that the average heritability of these environments was 27%. An individual’s genetically mediated behaviour impacts on their environmental; this is called gene-environment correlation (rGE), or the nature of nurture^21^. This rGE is especially interesting to explore in late adolescence given the changing social circumstances, which may allow individuals to have more freedom to select the support around them based on their genetic predispositions (resulting in increased heritability due to rGE) or these changing social circumstances may increase the influence of the external environment by pushing individuals into novel social contexts which they cannot control (resulting in decreased or stable heritability of our support measure in this age group).

To our knowledge, only one study has looked at the heritability of support quality in late adolescence^22^, which the authors invert and conceptualise as a proxy for social isolation, finding a heritability of 40%. Information is missing on the heritability of support quantity in adolescence as the rest of the genetically informative previous literature on support have all been conducted on adult or older adult samples. From these adult studies, heritability estimates for *quantity* of support are varied with some estimating there to be no heritable component^23,24^ while others estimating a large heritability of 75%^25^. The moderate levels of heritability estimates from the majority of adult studies to date suggest that rGE may be particularly important for these “environmental” social support constructs^21^. For many factors, the importance of genetic influences changes with age^26,27^. Scarr and McCartney^19^ proposed that the genetic influences on environmental measures would increase with development as individuals expand their experiences beyond the family environment and start to actively select their own environments. Given the changing nature of social circles during late adolescence, it was therefore important that we investigated the genetic influences on support quantity in our adolescent sample, in addition to trying to replicate the previous findings on support quality.

Another novel aspect of our study is that we include measures of both wellbeing and symptoms of depression (which we combine under the umbrella term of mental health), allowing us to investigate how social support is related to both. Wellbeing encompasses factors such as life satisfaction, emotional affect and happiness^28–30^. Based on a recent meta-analysis, wellbeing has a heritability of 36%^31^. Similar levels of heritability have been reported for happiness (33%) and life satisfaction (44%) in adolescence^32^. A large literature exists on the heritability of depression, with estimates ranging from 31–42%^33^, and at similar levels (40–45%) in adolescence^34^. It was important for us to conduct separate analyses for wellbeing and depression as the two are not 100% phenotypically correlated, with negative correlations of around −0.5 ^35^. It has been shown that individuals can have high levels of wellbeing and high levels of mental illness concurrently, as well as score low on both dimensions^36^. Furthermore, while they share some genetic overlap, the two also have genetic (and environmental) specificities^32,36^ suggesting the two to be aetiologically independent, but correlated, constructs.

Support and mental health have been found to be phenotypically associated in late adolescence^22,37,38^. One meta-analysis found that the association between support and mental health became stronger with increasing age from early childhood into late adolescence^16^ though this moderation effect was not replicated in a later meta-analysis^17^. Looking across a broad age range from early childhood to 20 years, both meta-analyses found perceived quality of support to be more strongly associated with mental health outcomes (better wellbeing and fewer depressive symptoms) than quantity of support.

As both social support and mental health are heritable, the two constructs may be phenotypically correlated due to a shared genetic aetiology. Only one study to date has investigated this genetic link in late adolescence^22^. The authors examined the genetic correlation between social isolation and depression in a sample of 18 year old UK twins. They found a genetic correlation of 0.33 and a non-shared environmental correlation of 0.15. The authors note that future studies should focus on incorporating measures that tap into other aspects of social networks. The only other study which has explored the genetic correlation between support and mental health was conducted on a Swedish sample of older adults^39^. Thus, we wished to expand on the work of these two studies, filling in the gaps within the literature by using another measure of social network, support quantity, and also linking both support quality and quantity with wellbeing (and related positive psychological traits) in addition to depression.

In our current study, using a sample of 18 year-old UK twins, we hypothesise that:

1. Both support quality and support quantity will be heritable.
2. There will be a moderate phenotypic link between these support measures with wellbeing and depression.
3. The association between support and mental health will be mediated by both genetic and environmental influences.

## Results

### Descriptive statistics

Observing the patterns of twin correlations (Supplementary Table S1) between MZ males and DZ males compared to MZ females and DZ females, we find evidence of potential quantitative sex differences, where the same genetic and environmental factors influence both males and females but to varying extents. DZ opposite sex twin correlations were generally lower than DZ same sex twin correlations which suggests qualitative sex differences, where there are different genetic and environmental influences on a trait for females and males. Our small sample sizes within each zygosity group meant that we were underpowered to run a sex-limitation model. Mean sex differences explained between 0.39% and 2.81% of the variance of our mental health and support measures (Supplementary Tables S2 and S3). Since the majority of the effects of sex were small, we have conducted analyses on the sample with males and females combined to maximise power. Our measures are also corrected for the mean effect of sex before conducting the twin model fitting analyses. For further information on sex differences in our sample, Supplementary Table S4 compares phenotypic correlations between mental health and support measures split by males and females belonging to same sex twin pairs. Mean difference between identical (monozygotic, MZ) and non-identical (dizygotic, DZ) twins was significant on the *significant other* subscale of support quality (p = 0.04), explaining 0.36% of the variance. When fitting our structural equation models, constraining our fully saturated models across twin order and zygosity did not significantly worsen the fit of our models (Supplementary Table S5) showing that the twin modelling assumptions of equal environments hold.

### Univariate Heritability Estimates

MZ intraclass correlations were all greater than DZ correlations suggesting that all the measures are genetically influenced (Table 1). We ran twin models to formally test the significance of genetic and environmental influences, and to provide confidence intervals. The sources of variation considered are additive genetic influences (A), non-additive genetic influences (D), shared environmental influences (C) and non-shared environmental influences (E). We ran ACE models for life satisfaction, support quantity and the *family* subscale of support quality because DZ correlations were more than half of the MZ correlations for these measures. We ran ADE models for the remaining measures because, for these, DZ correlations were less than half of the MZ correlations. We subsequently ran a series of reduced nested models to test the significance of the A, C/D and E parameters. Estimates of genetic and environmental influence on each trait are shown in Table 1 (based on the best fitting reduced AE model) and Supplementary Table S6 (full ACE/ADE model). Model fit statistics are shown in Supplementary Table S7 (including comparison of the full ACE/ADE model to a saturated model that makes no constraints on the data). Based on these fit statistics comparing nested models with the full model, it was shown that AE was the best fitting model for all constructs, apart from subjective happiness and relatedness. For these, the DE model had lower Akaike’s Information Criterion (AIC) values than the AE model, however neither models significantly worsened the fit compared to the full ADE model, at the significance level of 0.01. Since both models produced very similar fit statistics, and since A and D components are highly correlated, we decided to use the AE model for all constructs for our bivariate analysis.

**Table 1:** Monozygotic and Dizygotic Twin Correlations and Univariate Twin Analysis Results (with 95% confidence intervals).

	Monozygotic twin pairs N_pairs_ = 354	Dizygotic twin pairs N_pairs_ = 779	a^2^	e^2^
Positive Affect	0.49 (0.41, 0.57)	0.19 (0.12, 0.26)	0.47 (0.40, 0.54)	0.53 (0.46, 0.60)
Negative Affect	0.38 (0.29, 0.47)	0.15 (0.08, 0.21)	0.35 (0.27, 0.42)	0.65 (0.58, 0.73)
Subjective Happiness^1^	0.47 (0.39, 0.55)	0.16 (0.10, 0.23)	0.44 (0.36, 0.51)	0.56 (0.49, 0.64)
Life Satisfaction	0.57 (0.50, 0.64)	0.34 (0.28, 0.40)	0.60 (0.54, 0.65)	0.40 (0.35, 0.46)
Gratitude	0.58 (0.51, 0.65)	0.22 (0.15, 0.29)	0.55 (0.49, 0.61)	0.45 (0.39, 0.51)
Meaning in Life	0.53 (0.45, 0.60)	0.23 (0.16, 0.29)	0.52 (0.45, 0.58)	0.48 (0.42, 0.55)
Autonomy	0.52 (0.44, 0.60)	0.23 (0.16, 0.29)	0.51 (0.44, 0.57)	0.49 (0.43, 0.56)
Competence	0.51 (0.42, 0.58)	0.23 (0.16, 0.30)	0.50 (0.43, 0.56)	0.50 (0.44, 0.57)
Relatedness^1^	0.57 (0.50, 0.64)	0.21 (0.14, 0.28)	0.54 (0.47, 0.60)	0.46 (0.40, 0.53)
Depression	0.41 (0.32, 0.50)	0.20 (0.13, 0.26)	0.40 (0.33, 0.47)	0.60 (0.53, 0.67)
Support quantity	0.49 (0.41, 0.57)	0.27 (0.21, 0.34)	0.49 (0.43, 0.55)	0.51 (0.45, 0.57)
Support quality (*total*)	0.55 (0.48, 0.62)	0.27 (0.20, 0.33)	0.55 (0.48, 0.60)	0.45 (0.40, 0.52)
Support quality (*significant other*)	0.44 (0.35, 0.52)	0.21 (0.15, 0.28)	0.43 (0.36, 0.50)	0.57 (0.50, 0.64)
Support quality (*family*)	0.59 (0.52, 0.65)	0.31 (0.25, 0.37)	0.59 (0.53, 0.64)	0.41 (0.36, 0.47)
Support quality (*friends*)	0.40 (0.31, 0.49)	0.15 (0.08, 0.22)	0.37 (0.29, 0.44)	0.63 (0.56, 0.71)

*Note*. The univariate results are from the best fitting (AE) model with variance components due to additive genetic (a^2^) and non-shared environmental (e^2^) effects.

^1^While both AE and DE model fit statistics were extremely similar, the DE model had lower AIC values than the AE model for subjective happiness and relatedness. Running the DE for these two constructs produced the following results: Subjective happiness d^2^ = 0.49 (0.41, 0.56) and e^2^ = 0.51 (0.44, 0.59), Relatedness d^2^ = 0.58 (0.51, 0.63) and e^2^ = 0.42 (0.37, 0.49).

Based on the best-fitting AE model, the heritability of quality of support was 55% (95% CI = 48%, 60%) and of support quantity was 49% (95% CI = 43%, 55%). Examining the subscales for support quality, the *family* subscale showed the highest heritability (a^2^ = 59%, 95% CI = 53%, 64%). Heritability estimates for mental health measures were moderate, with the lowest for negative affect (a^2^ = 35%, 95% CI = 27%, 42%) and the highest for life satisfaction (a^2^ = 60%, 95% CI = 54%, 65%).

### Phenotypic Correlations

**Figure 1:**
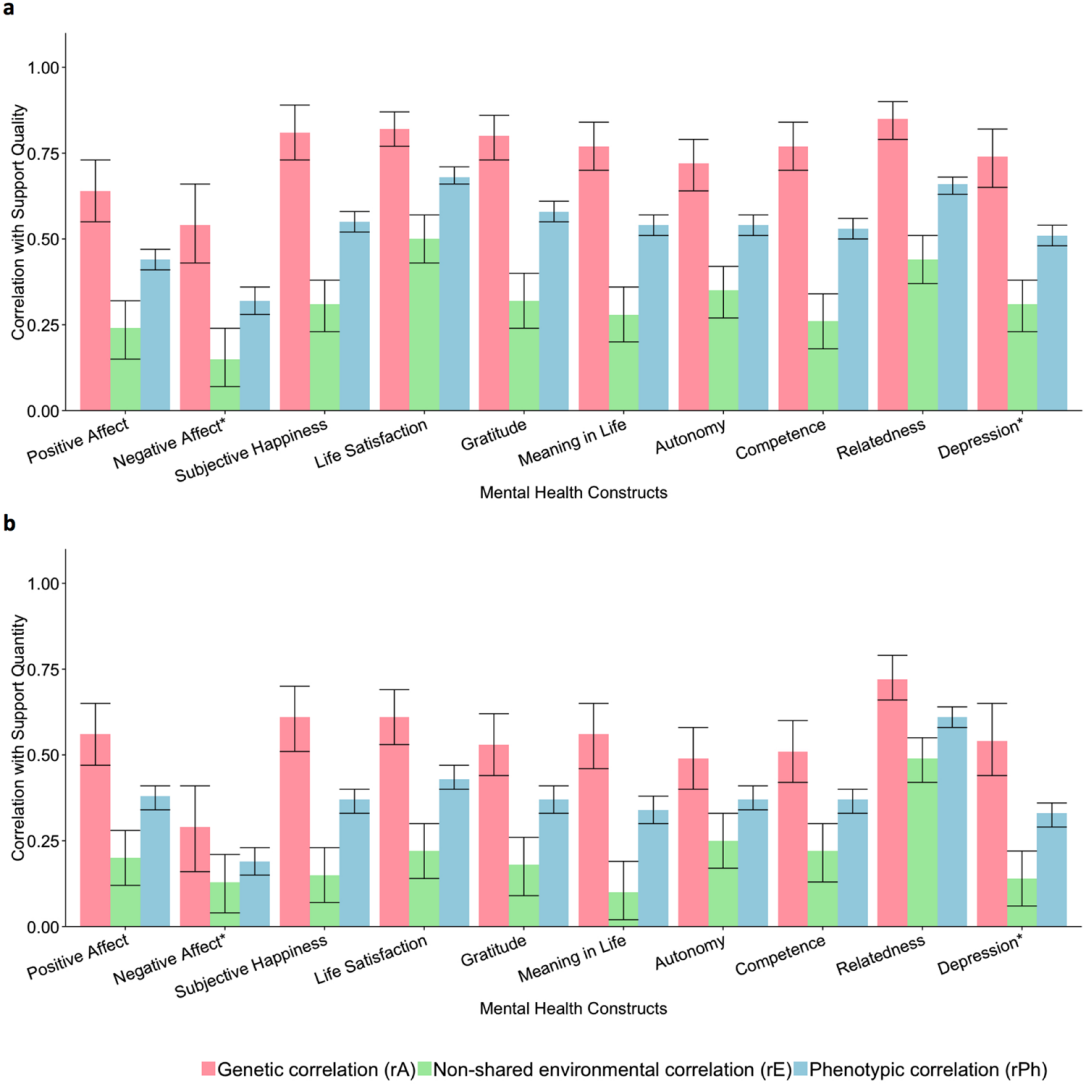
Genetic, non-shared environmental and phenotypic correlations with 95% confidence intervals. The results here are obtained from bivariate AE model fitting using Cholesky decomposition, presented in the form of a mathematically equivalent correlated factors solution, which provides estimates of genetic and environmental correlations between our measures. All correlations were in the expected direction with higher levels of both support quality and quantity associated with higher levels of wellbeing and lower levels of depression. *For comparative purposes, correlations with negative affect and depression shown here are absolute, as measures are negatively correlated with both support measures **(a**) Correlations between support quality (total score) and mental health constructs. Absolute average phenotypic correlations between total support quality score and mental health = 0.54. Absolute average genetic correlation = 0.75, absolute average non-shared environmental correlation = 0.32. **(b)** Correlations between support quantity and mental health constructs. Absolute average phenotypic correlations between support quantity score and mental health = 0.38. Absolute average genetic correlation = 0.54, absolute average non-shared environmental correlation = 0.21.

Phenotypically, quality of support and quantity of support had a correlation of 0.51 (95% CI = 0.48, 0.54). The *friends* subscale of support quality had the highest phenotypic correlation with support quantity, which was to be expected as the support quantity questionnaire only asked questions related to friends (Table 2). Phenotypic correlations between the mental health measures ranged (in absolute size) from 0.15 (95% CI = 0.11, 0.20) between positive affect and negative affect (negative correlation) to 0.71 (95% CI = 0.69, 0.74) between competence and meaning in life (Table 3). Given the variability in phenotypic correlations between these measures, we decided to conduct bivariate analyses between mental health and support separately for the different mental health constructs. We also believe this separation will be more informative for future studies that are unlikely to have the exact same set of wellbeing indicators. The phenotypic correlations between the support and mental health scales were all in the expected direction with higher levels of support correlated with higher levels of wellbeing and lower levels of depressive symptomatology (Fig. 1 and Table 2; absolute mean correlation = 0.46). Phenotypic correlations between total support quality and mental health were all higher (absolute mean = 0.54) than between support quantity and mental health (absolute mean = 0.38). All subscales of support quality tended to have lower correlations with the mental health measures than the total combined score. Often, the higher correlations of the total combined score were driven by higher correlations between the mental health measure and the *family* subscale (such as for subjective happiness and life satisfaction) or between the mental health measure and the *friend* subscale (such as for relatedness). The *significant other* subscale of support quality tended to be less important for most of the mental health measures.

**Table 2:** Phenotypic Correlations between Mental Health and Support Constructs (95% Confidence Intervals).

	Support quantity	Support quality (*total*)	Support quality (*significant other*)	Support quality (*family*)	Support quality (*friends*)
Positive affect	0.38 (0.34, 0.41)	0.44 (0.41, 0.47)	0.36 (0.32, 0.40)	0.37 (0.33, 0.41)	0.36 (0.32, 0.39)
Negative affect	−0.19 (−0.23, −0.15)	−0.32 (−0.36, −0.28)	−0.19 (−0.24, −0.15)	−0.33 (−0.37, −0.29)	−0.27 (−0.31, −0.23)
Subjective happiness	0.37 (0.33, 0.40)	0.55 (0.52, 0.58)	0.41 (0.38, 0.45)	0.49 (0.46, 0.52)	0.45 (0.41, 0.48)
Life satisfaction	0.43 (0.40, 0.47)	0.68 (0.66, 0.71)	0.48 (0.44, 0.51)	0.63 (0.61, 0.66)	0.59 (0.56, 0.61)
Gratitude	0.37 (0.33, 0.41)	0.58 (0.55, 0.61)	0.45 (0.41, 0.48)	0.57 (0.53, 0.59)	0.43 (0.39, 0.46)
Meaning in life	0.34 (0.30, 0.38)	0.54 (0.51, 0.57)	0.44 (0.40, 0.47)	0.48 (0.45, 0.52)	0.42 (0.38, 0.45)
Autonomy	0.37 (0.34, 0.41)	0.54 (0.51, 0.57)	0.38 (0.34, 0.42)	0.50 (0.47, 0.54)	0.45 (0.42, 0.49)
Competence	0.37 (0.33, 0.40)	0.53 (0.50, 0.56)	0.40 (0.37, 0.44)	0.48 (0.45, 0.51)	0.42 (0.38, 0.45)
Relatedness	0.61 (0.58, 0.64)	0.66 (0.63, 0.68)	0.48 (0.44, 0.51)	0.49 (0.46, 0.53)	0.66 (0.63, 0.68)
Depression	−0.33 (−0.36, −0.29)	−0.51 (−0.54, −0.48)	−0.36 (−0.40, −0.33)	−0.48 (−0.52, −0.45)	−0.41 (−0.45, −0.38)
Support quantity	1				
Support quality (*total*)	0.51 (0.48, 0.54)	1			
Support quality (*significant other*)	0.34 (0.31, 0.38)	0.85 (0.84, 0.86)	1		
Support quality (*family*)	0.27 (0.23, 0.31)	0.80 (0.78, 0.82)	0.53 (0.50, 0.56)	1	
Support quality (*friends*)	0.65 (0.63, 0.68)	0.81 (0.80, 0.82)	0.55 (0.52, 0.58)	0.46 (0.42, 0.49)	1

*Note*. Phenotypic correlations were obtained through full information maximum likelihood (FIML) from the constrained saturated bivariate model. Our bivariate saturated model, which constrained means across sex and age, included within-twin within-trait, within-twin cross-trait, cross-twin within trait and cross-twin cross trait covariances. Our bivariate saturated model was constrained across twin order and zygosity to give us phenotypic correlation estimates between our measures for the population with confidence intervals. The FIML method handles missing data. Total number of observations (twin pairings) used in each analysis was N = 1,133.

**Table 3:** Phenotypic Correlations between Mental Health Constructs (95% Confidence Intervals).

	Positive Affect	Negative Affect	Subjective Happiness	Life satisfaction	Gratitude	Meaning in Life	Autonomy	Competence	Relatedness
Negative Affect	−0.15 (−0.20, −0.11)								
Subjective Happiness	0.50 (0.46, 0.53)	−0.48 (−0.52, −0.45)							
Life satisfaction	0.47 (0.43, 0.50)	−0.48 (−0.51, −0.45)	0.66 (0.64, 0.69)						
Gratitude	0.49 (0.46, 0.53)	−0.34 (−0.38, −0.31)	0.54 (0.51, 0.57)	0.60 (0.57, 0.62)					
Meaning in Life	0.62 (0.60, 0.65)	−0.37 (−0.41, −0.34)	0.57 (0.54, 0.60)	0.60 (0.57, 0.62)	0.61 (0.58, 0.64)				
Autonomy	0.42 (0.39, 0.46)	−0.47 (−0.51, −0.44)	0.54 (0.51, 0.57)	0.57 (0.54, 0.6)	0.51 (0.48, 0.54)	0.56 (0.53, 0.59)			
Competence	0.59 (0.56, 0.61)	−0.43 (−0.46, −0.39)	0.54 (0.51, 0.57)	0.58 (0.55, 0.61)	0.54 (0.51, 0.57)	0.71 (0.69, 0.74)	0.65 (0.62, 0.67)		
Relatedness	0.47 (0.43, 0.50)	−0.39 (−0.43, −0.35)	0.56 (0.53, 0.59)	0.63 (0.61, 0.66)	0.61 (0.58, 0.63)	0.57 (0.54, 0.59)	0.65 (0.63, 0.68)	0.63 (0.60, 0.65)	
Depression	−0.48 (−0.51, −0.45)	0.62 (0.59, 0.65)	−0.61 (−0.64, −0.59)	−0.64 (−0.66, −0.61)	−0.53 (−0.56, −0.49)	−0.59 (−0.62, −0.56)	−0.55 (−0.58, −0.52)	−0.58 (−0.60, −0.55)	−0.55 (−0.58, −0.52)

### Bivariate Heritability Estimates

We fitted bivariate AE models to explore the relationship between the two support measures and between mental health and support. Support quantity had a genetic correlation with total support quality of 0.64 (95% CI = 0.56, 0.71), and an environmental correlation of 0.38 (95% CI = 0.30, 0.45). These correlations were mainly driven by the *friends* subscale of support quality (Supplementary Table S8).

**Figure 2:**
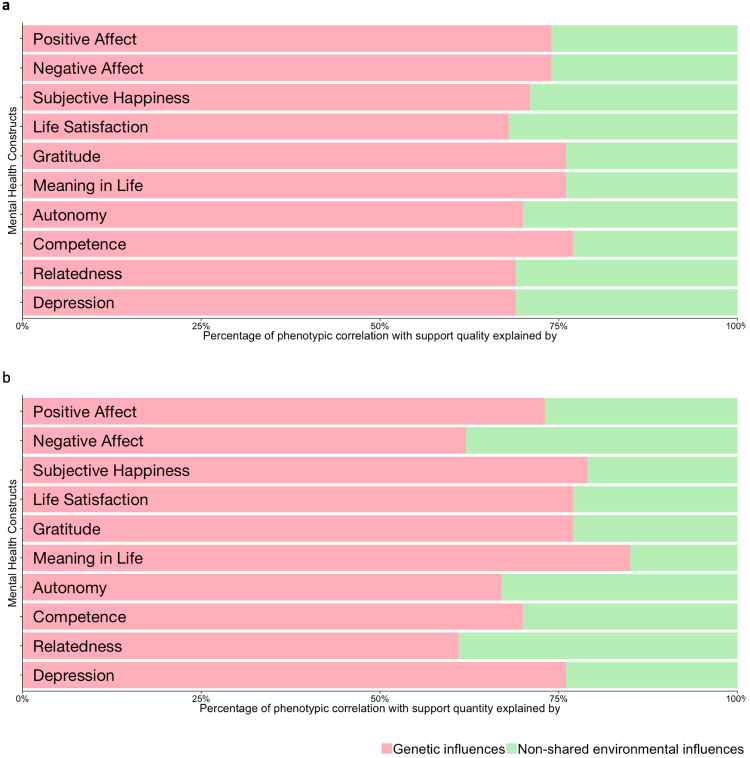
Bivariate AE model fitting results for support measures and mental health constructs. The results here are from a bivariate Cholesky decomposition, presented in the form of a mathematically equivalent correlated factors solution, enabling us to calculate the proportion of the phenotypic correlation between our measures of mental health and social support that is accounted for by overlapping genetic or environmental influences. Genetic influences explained a larger proportion of the phenotypic correlations between all of the measures of mental health and support. This applied to both **(a)** support quality and **(b)** support quantity.

Figure 1 shows the genetic and environmental correlations (see also Supplementary Table S8) between the support and mental health measures. Most of the genetic correlations were moderate to high (absolute mean rA = 0.67, absolute range = 0.29 to 0.85), while the non-shared environmental correlations tended to be much lower (absolute mean rE = 0.25, absolute range = 0.07 to 0.53). Common genetic influences explained a larger proportion of the phenotypic correlations between all of the measures of mental health and support (mean proportion of correlation explained by genetic overlap = 72%, range = 57% to 85%) than common environmental influences (Fig. 2 and Supplementary Table S9).

As with the phenotypic correlations, genetic and non-shared environmental correlations were all greater for support quality with the mental health outcomes than for support quantity (Fig. 1 and Supplementary Table S8). Differences across the support quality subscales could be seen in the genetic and environmental correlations with the mental health measures (Supplementary Table S8), which reflected the phenotypic results. For example, correlations were lower for the *significant other* subscale with negative affect and life satisfaction. Correlations were noticeably higher between the *friends* subscale and relatedness and between the *family* subscale and gratitude. Due to the potential overlap in item content across the subscales, namely due to items in the *significant other* subscale such as “There is a special person who is around when I am in need”, we believed it was best to focus our results on the overall combined support quality scale.

Bivariate results were comparable across our range of wellbeing measures, and followed the same trend as our depression measure. Interestingly, life satisfaction had higher phenotypic correlations with support quality (r = 0.68, 95% CI = 0.66, 0.71), compared to all other mental health constructs, with non-overlapping confidence intervals, other than relatedness. While genetic correlations between life satisfaction and support quality were not different to other constructs, the higher phenotypic correlation was due to the larger non-shared environmental correlation (rE = 0.50, 95% CI = 0.43, 0.57).

## Discussion

The first aim of this study was to examine the magnitude of genetic and environmental influences on two types of social support in late adolescence. We found the heritability of support quality and quantity to be 55% (95% CI = 48%, 60%) and 49% (95% CI = 43%, 55%) respectively. Our heritability estimate for support quality (55%) is similar to the estimate of 40% (95% CI = 34%, 46%) for social isolation (the inverse of the same measure that we used for support quality) found in a similar sample of 18 year old UK twins^22^. The slight difference in estimates could be due to sample differences, as participants in our sample were selected if they owned a social media account (Twitter), which is likely to indicate genetic predispositions associated with social interaction (possibly increasing rGE). We add to the literature by showing that support quantity at this age is also moderately heritable, and this estimate is much higher than those found in some previous studies looking at older adults^23^. This high heritability of support quantity in adolescence is in line with one of the most prominent social network theories – the socioemotional selectivity theory^40^ which posits that having a large social network is a much more salient priority for young people. This priority is perhaps driving young people to actively seek out and select new acquaintances and social experiences, and this is possibly resulting in the higher heritability of support quantity in adolescents compared to older adults, again through rGE. Indeed, global social networks have been shown to increase during adolescence into young adulthood, before shrinking in later adulthood^8^.

There are three mechanisms by which genetically influenced characteristics of the individual could influence the support environment^19^. Parents provide both genes and the shared environment for the individual that results in passive gene-environment correlation (rGE). Active rGE occurs when individuals seek out experiences based on their genotype – people could be seeking supportive network sizes that match their genetic preference (resulting in the heritability of support quantity) or seeking certain types of friends who differ in the type and quality of support they provide (resulting in the heritability of support quality). Evocative rGE occurs when an individual’s genetic characteristics evoke certain reactions from others – for example, genetically mediated traits, such as personality, could elicit more supportive behaviour from others or cause other people to want to come into more frequent contact with them. Undoubtedly, a large combination of genetic characteristics will be involved, and our design is unable to unequivocally separate these different mechanisms of rGE.

Phenotypic correlations between support quality and support quantity were moderate and slightly higher than the weak associations that have previously been found^41^. In relation to our second hypothesis, we also found moderate phenotypic correlations between mental health and our support measures in our late adolescent sample, that are higher than those found in adult samples^42,43^ and comparable with the correlation between depression and social isolation found by Matthews and colleagues in their late adolescent sample^22^. Individuals with better social support had higher wellbeing and fewer symptoms of depression. Our cross-sectional analysis was unable to elucidate the question of causality, but future cross-lagged longitudinal studies could give us such insight.

The third aim of our study was to explore the degree to which genetic and environmental influences can account for the relationship between social support and mental health. We found genetic correlations between our support and mental health measures to be moderate, with genetic influences explaining most of the phenotypic correlation between them. Our genetic correlation between depression and support quality of −0.60 is higher than that found by Matthews and colleagues of 0.33^22^ (they used the inverse of support quality). Differences are likely to be due to our self-report measure of depression using CES-D compared to their interview measure using the Diagnostic Interview Schedule. The environmental correlations were very comparable (0.15 and 0.19 respectively). Our measure of life satisfaction allowed us to compare our results with the only other study that has looked at the genetic correlation between wellbeing and support. In an older adult Swedish sample (mean age = 65.6 years), genetic influences were found to explain 56% of the phenotypic correlation (r = 0.27) between perceived support and life satisfaction^39^. We found genetic influences to explain 68% of the phenotypic correlation (r = 0.65) between perceived quality of support and life satisfaction. So interestingly, support and life satisfaction are much more correlated in our younger sample, and genetic influences explain slightly more (68% versus 56%) of the higher phenotypic correlation. Further studies should use longitudinal twin models to give us more insight into the connection between support and wellbeing over the lifecourse. Furthermore, our results using life satisfaction gave interesting insights into the difference between wellbeing and depression, even showing a special relationship with support quality that is not seen in the other wellbeing measures, perhaps indicating a unique non-shared environmental pathway. Further studies should try to find the non-shared environments that are distinctively driving the higher phenotypic correlation between life satisfaction and support quality. More generally, this provides even more evidence for the utility of using wellbeing in giving us insights that would not have been found from looking at depression alone.

Based on our findings, we are unable to infer whether support or mental health is primary to the other, just that they share a genetic aetiology. Alternatively, the genetic relationship could be explained by genetic influences on a third variable, such as personality, which impacts on both mental health and support. The crucial implication of this finding is that we should not think of social support as a purely environmental experience that has a direct causal influence on our mental health outcomes. Shared genetic influences are important in explaining the relationship between social support and mental health. Studies of phenotypic correlation which do not consider gene-environment correlations are in danger of providing biased estimations of the size of the effect the “environmental” measure (social support) has on the outcome (mental health). Experiences, like social support, do not just passively happen to us; we create, select, seek out and perceive our social support partly based on our genetic predispositions. Simply changing the social support available to individuals will not necessarily improve their mental health.

The environmental correlations were all substantially lower than the genetic correlations. However, their absolute magnitudes were all greater than 0 and were all in the expected direction. This indicates that there are also some non-shared environmental influences that cause both increases in wellbeing (and decreases in depression) and increases in support quality and quantity, though the effect is much smaller than the effect of shared genetic influences. As an example, one of these non-shared environmental factors could be a late adolescent’s extra-curricular activities. These activities will create unique environments which can have an impact on the social circles formed, since extra-curricular activities are one of the primary ways through which young people maintain existing, and develop new friendships^44^. Concurrently, these extra-curricular activities could also have an impact on an individual’s mental health^45^ that could be mediated by mechanisms that are independent of relationships, for example through identity formations^46^. Of course, many of these unique environments selected will themselves be under genetic influence, due to an individual’s genetically predisposed preferences. This will even incorporate life events such as being in a car accident or hospitalisation^47,48^. Our study is unable to identify specific environments driving the phenotypic correlation between support and mental health, though methods such as the MZ differences design could help us identify such factors that are independent of genetic influences^49,50^.

Our phenotypic results follow a similar pattern to previous studies which have found stronger links between the quality (in contrast to quantity) of support and mental health outcomes in both adults^24,42,43,51^ and children^16,17^. We add additional weight to the evidence by finding that genetic correlations are also higher between support quality and mental health in late adolescence. While loneliness is always associated with negative outcomes, namely depression^52,53^, solitude, or a retreat from social contact, may in itself be a positive experience^54^. Solitude has been associated with better adjustment and emotional state^55,56^. Furthermore, individual differences have been shown to exist in the preference for solitude^57^. Therefore, one explanation that we propose for the difference between quality and quantity of support in their relation to mental health is that while most individuals experience benefits to their mental health by knowing that they have good quality support around them, there are individual differences in the desired frequency of actual supportive contact. It would be useful for future studies to examine the positive benefits an individual may experience when their preference for solitude and their experience of the social environment “fits” (similar to Lyubomirsky and Layous’ Positive Activity Model^58^ from the wellbeing intervention literature). It will be important to assess whether quantity and quality have different relationships with mental health based on context. For example, when an individual is going through a period of stress quantity may have a higher correlation with mental health compared to when an individual is no longer in distress when this correlation may be lower. This is crucial because it will inform the personalisation of preventative measures based on the environmental conditions an individual is facing. However, we must remember that our two measures of support were moderately correlated, suggesting that, for many individuals, quantity *is* related to quality of social contact, though this relationship may not necessarily be linear. Furthermore, reporting biases may be at play: individuals who have a more positive outlook in life will rate their support as more satisfactory and also report being mentally healthier. This effect may be stronger for our support quality measure since this requires more subjective appraisal while reporting frequencies in our support quantity measure may be less under the influence of appraisal bias.

Overall, our results suggest that social support and mental health are related in late adolescence, and that shared genetic factors mediate this association. The higher correlations seen between support quality and mental health, in contrast to support quantity suggests that interventions could focus on helping young people to be more aware of the support that is available to them, as changing the objective environmental condition may not be as effective for everyone. There is also scope for personalising interventions to match personal characteristics with different types of social support interventions (e.g. increasing social network size versus increasing awareness of support).

Within the support quality measure, our results also suggested that family and friends tended to be more important for mental health than the support from a significant other. This is understandable as romantic relationships are only starting to form at this age. Furthermore, this ties in with the patterns seen in the life-course development of social relationships, in which supportive network sizes are larger in adolescence than adulthood^8^, suggesting the importance of larger supportive circles over one close relationship.

Our study has many strengths, including a focus on an interesting developmental stage, the use of mental health measures of both wellbeing and depression, and social support measures of both quantity and quality. However, there are ways in which the data we have available are limited. All of our measures were self-reported, meaning they all have a degree of subjectivity. Especially when considering perceived support quality and mental health, the link may be due to a positive attribution style or reporting bias based on individual personality or reporting style, which is a limitation of the use of self-report questionnaires. Focussed studies of social support should attempt to collect a combination of subjective and objective ratings of social support networks through the use of multiple raters, peer nominations and independent observers. In our exploratory analysis, we found evidence to suggest there are quantitative and qualitative sex differences for some of our measures, which partly aligns with previous genetically informed sex differences studies^59,60^. Phenotypic correlations between support and mental health, split by sex, also suggested a stronger link in females which aligns with previous phenotypic studies^61^. We were limited by the sample size of our data and so were underpowered to run full sex-limitation models but future well-powered studies should focus on exploring these sex differences. In addition, we find small but statistically significant differences in the demographics between the subsample of TEDS who participated in the current study and the complete TEDS sample (Table 4). These differences seem in line with the demographics of social media users^62,63^ but we cannot rule out the possibility of selection biases and the possibility of differences between social media users and non-users, and survey responders and non-responders.

**Table 4:** Representativeness of Sample

	Original TEDS sample	16 year TEDS sample	Social Networks Questionnaire Invitation sample	Social Networks Questionnaire Response sample	χ^2^ test/ t-test	p-value
N families	13,734 families	7,723 families	1547 families	1215 families (78.5% response rate)	—	—
% Female	50.24%	52.45%	60.85%	63.41%	133.53	6.91e-31***
% MZ	33.34%	35.13%	31.71%	30.52%	26.19	3.09e-07***
% White	91.70%	92.98%	93.59%	94.88%	15.59	7.88e-05***
Mean SES composite (SE)	0.00 (1.00)	0.17 (0.99)	0.16 (0.94)	0.19 (0.93)	1.12	0.26
Mean Life Satisfaction (SE)	—	5.69 (1.07)	5.67 (1.09)	5.69 (1.09)	0.32	0.75
Mean Depression (SE)	—	3.62 (4.45)	3.87 (4.69)	3.79 (4.62)	1.56	0.12

*Note*. Table showing representativeness of sample of TEDS participants who took part in the current study. ***p < 0.001. % female relates to the sex of the individuals. MZ = monozygotic twins. % White = ethnicity of individual reported at first contact. Socioeconomic status (SES) composite is composed of 5 derived variables relating to parent qualifications and employment, and mother’s age at birth of first child, and is standardised at first contact. Life satisfaction is a composite score (ranging from 1 to 7) from the Brief Multidimensional Students’ Life Satisfaction Scale (BMSLSS), measured at age 16. Depression is a composite score (ranging from 0 to 26) from the Moods and Feelings Questionnaire, measured at age 16.

Original TEDS sample refers to the information of all the individuals who were first contacted to take part in the study between 1994 and 1996. 16 year TEDS sample refers to all individuals who completed the main TEDS data collection between 2010 to 2012, when participants were roughly 16 years old. During this data collection, participants completed comprehensive mental health questionnaires, including life satisfaction and depression. Social Networks Questionnaire Invitation sample refers to all the individuals who were invited to take part in the current study, and these invited individuals were selected based on previous indication that they (or their co-twin) used online social media (e.g. Twitter or Facebook). Social Networks Response sample refers to all individuals who completed and returned the postal questionnaire used in this current study. 32 families completed and returned this postal questionnaire but did not complete the data collection in the full 2010–2012 study. Of the participants who took part in the main 2010–2012 data collection, χ^2^ tests/t-tests were used to statistically compare proportions/means between participants who went on to complete the current study and participants who did not go on to take part in the current study.

As with all statistical analyses, the twin design makes several assumptions. In general, these assumptions have been tested and appear reasonable, for example the equal environments assumption in which monozygotic and dizygotic twins share the same environment to the same extent^64,65^ and the assumption of generalisability of twin results to the rest of the population^66–68^. In our model fitting analyses we found that the AE model was the best-fitting model. However, we could be underpowered to detect the smaller effects of C or D as significant. One implication of this is that we may be inflating the importance of genetic influences (by dropping C in our reduced model) or deflating it (by dropping D). However, when we compare the overall genetic influence from the full model (Supplementary Table S2) to the estimate of additive genetic influence in the reduced AE model (Table 1), we find very similar levels of heritability. Future studies with larger sample sizes would be better placed to estimate the separate influence of additive and non-additive genetic factors, but, as A and D are highly correlated, it is unlikely our results would appear very different.

A future direction would be to determine if the same genetic and environmental influences on support that are important in youth continue to have the same effect into later adulthood, or if new genetic and environmental factors come into play. Furthermore, we could not infer causation from our analysis. Longitudinal twin models could help us answer both these questions. As increasingly well-powered molecular genetic studies give us more detailed information about the individual genetic variants that influence complex traits, we will also be able to use approaches such as Mendelian Randomisation^69^ to infer causation.

In summary, we found that in our late adolescent sample, genetic and environmental influences were equally important in explaining individual differences in both support quality and support quantity. The heritability of support could be due to a combination of passive, active and evocative gene-environment correlations in which the genetic predisposition of individuals (and their parents) dictate, alter and reconstruct the external environment of support. Individuals with higher levels of social support had better mental health, although support quality had stronger links than support quantity, and these associations were mediated primarily by shared genetic influences. We also found that genetic correlations were higher between support quality (compared to support quantity) and mental health. We speculate that while most individuals will experience benefits to their mental health by knowing that they have good quality support around them, there are individual differences in the desired frequency of actual supportive contact.

## Methods

### Sample

Participants were a subsample of a larger population cohort, the Twins Early Development Study (TEDS)^70^. Born in England and Wales between 1994 and 1996, TEDS twins have been followed throughout childhood into adulthood. At first contact, when the twins were 18 months old, a parent-report questionnaire of physical similarity^71^ was used to assign zygosity, with DNA tested if zygosity was unclear from the questionnaire alone.

Data for this study were collected as part of a project on online social networks in late adolescence. Participants from the main TEDS sample were invited to complete this study if they (or their co-twin) had previously indicated that they used online social media (e.g. Twitter or Facebook). Ethical approval was provided by the Institute of Psychiatry research ethics committee at Kings College London (Ref: PNM/12/13–34) and informed consent was obtained from all participants. All methods were carried out in accordance with relevant guidelines and regulations. 1,215 twin pairs (78.5% of those invited to take part) completed a postal questionnaire that contained measures of social support and mental health. Of these, 57 twin pairs were excluded, either due to missing zygosity information (N = 6 twin pairs) or birth complications, autism, or medical conditions (N = 51 twin pairs). After these exclusions the sample included 1,158 twin pairs who provided relevant data for the final analysis. The mean age of the sample was 17.85 (SD = 0.77) and 64% were female. 31% of the sample were MZ twins and 69% were DZ twins. Of these DZ twins, 51% were same-sex pairs, and 49% were opposite-sex pairs. Supplementary Tables S2 and S3 provides more information about the composition of the sample, split by sex and zygosity for each of the measures of interest. The representativeness of this subsample is presented in Table 4 and compared with data for the whole TEDS sample. In general, the current subsample is reasonably representative of the whole TEDS sample. There was a slight tendency for this sub-sample to have proportionally more white participants and proportionally less MZ twin pairs, but the magnitude of these differences was quite small. This sub-sample does have more female participants than the main TEDS sample (63% versus 52%), which is likely driven by the higher uptake of social network use in females compared to males^62,63^.

### Measures

Details of all measures used in our analysis are shown in Table 5, including information on the number of items in each scale, item scoring, and an example item. In total, we used nine measures of wellbeing, one measure of depression and two measures of social support. Our measure of support quality is the Multidimensional Scale of Perceived Social Support. This scale is formed from a combination of three subscales, perceived support from family, from friends and from a significant other, and we conducted supplementary analysis on these three subscales in addition to our main analysis using the total combined scale. Our measure of support quantity is the Lubben Social Network Scale – Revised. The original measure includes two subscales, one for family and one for friendships. We included just the friendship subscale of this measure because for young people who are still living with their families there is limited variation in the frequency that they see their relatives (i.e. most participants would see a family relative everyday). Therefore, we were unable to conduct any subscale analysis using this measure. All measures demonstrated good internal consistency in our sample (Table 5).

**Table 5:** Description of measures.

**Construct**	**Measure Name**	**Number of items**	**Sample item**	**Item scoring**	**Higher score represents**	**Internal consistency**, α
*Social Support*						
Support quantity	Lubben Social Network Scale – Revised (friendships section)^82^	6	“How many friends do you see or hear from at least once a month?”	6 point scale from “none” to “nine or more”, or from “less than monthly” to “daily”, or from “never” to “always”	Greater quantity of support	0.82
Support quality	Multidimensional Scale of Perceived Social Support^83^	12	Family subscale (4 items): “My family really tries to help” Friend subscale (4 items): “I can count on my friends when things go wrong” Significant other subscale (4 items): “There is a special person who is around when I am in need”	7 point scale from “very strongly disagree” to “very strongly agree”	Greater quality of support	0.93
*Wellbeing*						
Positive affect	Positive and Negative Affect Scale (PANAS) positive affect subscale^84^	10	“Indicate to what extent you have felt this way during the past few weeks: Interested”	5 point scale from “very slightly or not at all” to “extremely”	Greater positive emotion	0.88
Negative affect	Positive and Negative Affect Scale (PANAS) negative affect subscale^84^	10	“Indicate to what extent you have felt this way during the past few weeks: Distressed”	5 point scale from “very slightly or not at all” to “extremely”	Less negative emotion	0.86
Subjective happiness	Subjective Happiness Scale^85^	4	“Some people are generally very happy and enjoy life regardless of what is going on. To what extent does this describe you?”	7 point scale from “not at all” to “a great deal”	Greater happiness	0.81
Life satisfaction	Brief Multidimensional Students’ Life Satisfaction Scale^86^	6	“How happy are you with your friendships?”	7 point scale from “very dissatisfied” to “very satisfied”	Greater life satisfaction	0.84
Gratitude	Gratitude Questionnaire^87^	6	“I have so much in life to be thankful for.”	7 point scale from “strongly disagree” to “strongly agree”	Higher level of gratitude	0.83
Meaning in life	Meaningful life scale^88^	5	“My life interests and excites me.”	7 point scale from “strongly disagree” to “strongly agree”	Higher level of meaning in life	0.86
Autonomy	Basic Psychological Needs Scale autonomy subscale^89,90^	7	“I feel like I am free to decide for myself how to live my life”	7 point scale ranging from “not at all true” to “very true”	Higher level of autonomy	0.71
Competence	Basic Psychological Needs Scale competence subscale^89,90^	6	“Often I do not feel very competent”	7 point scale ranging from “not at all true” to “very true”	Higher level of competence	0.77
Relatedness	Basic Psychological Needs Scale relatedness subscale^89,90^	8	“I really like the people I interact with”	7 point scale ranging from “not at all true” to “very true”	Higher level of relatedness	0.84
*Depression*						
Depression	Centre for Epidemiologic Studies Depression Scale^91^	20	“I was bothered by things that usually don’t bother me”	4 point scale from “rarely or none of the time” to “most or all of the time”	Lower level of depressive symptomology	0.88

### Data Preparation

For all measures, composites were created by taking the mean of the items (requiring 50% of the items to be non-missing). This is standard procedure for all composite formation in TEDS. Raw scores were used for our descriptive analysis and scores were subsequently standardised for our twin analyses. In addition, as is standard in twin analysis, all measures were corrected for the mean effects of age and sex^72^. As expected, several measures were skewed (towards better wellbeing, fewer symptoms of depression, and good social support) (Supplementary Table S10). Because OpenMx’s full-information maximum likelihood procedure is fairly robust to non-normality, we decided to conduct the analyses on untransformed scales. However, for those measures with a skew over 1, we repeated the analyses on a reflected then log-transformed version of those scales with negative skew and directly log-transformed version of those scales with positive skew. Results using transformed scales (presented in Supplementary Figures S11–S21) showed a very similar pattern of findings, with confidence intervals that overlapped with the untransformed analyses.

### Twin Analyses

Comparing intraclass correlations^73^ of MZ and DZ twins allows us to estimate the relative contributions of genetic and environmental influences that cause variation in a trait within a population^18^. The sources of variation considered are additive genetic influences (A) which indicates the total effect of an individual’s alleles which have additive effects on the trait, non-additive genetic influences (D) which are most often due to interactions between alleles at the same locus (dominance effect) or between alleles at different loci (epistatic effects), shared environmental influences (C) which make members of a family similar to one another, and non-shared environmental influences (E) which make twins different from each other and which includes measurement error.

We used structural equation model fitting to formally test our twin models^74^. The OpenMx 2.0 package^75–77^ in R Studio (Version 1.0.136)^78,79^ was specially developed for this type of analysis. Covariance models are fitted to the raw data and latent genetic and environmental factors, in addition to the observed factors, are estimated using full-information maximum likelihood. Goodness of fit was assessed using log likelihood comparisons and AIC values.

### Fitting the Univariate Model

A diagram of the basic univariate twin model is shown in Supplementary Figure S22. The total phenotypic variance of a trait is a sum of A, D, C and E. A small proportion of the human genome varies from individual to individual. Focussing specifically on these variants, MZ twins are 100% genetically identical while DZ twins are on average 50% genetically identical. We assume that both reared together MZ and DZ twins share 100% of their shared environment (C), while MZ twins share 100% of D while DZ twins share 25% of D (Supplementary Figure S23). Using this information of genetic and environmental similarity, we can predict concordances of a trait between MZ and DZ twin pairs and compare this to actual observed concordances to enable us to decompose the phenotypic variance of a trait using simultaneous equations (displayed in Supplementary Figure S23). When MZ twin correlations are greater than DZ twin correlations, we can expect genetic factors to be important for the trait. In the classic twin model, only one of D and C can be estimated at any one time. Considering the genetic relatedness between the twins, ACE models are used when DZ correlations are more than half of the MZ correlation since C is suggested. ADE models are used when the DZ correlation is less than half of the MZ correlation^74^. When MZ twins are less than 100% phenotypically correlated this indicates the importance of E for the trait.

Firstly, we fit “full univariate models” which contain all the possible sources of variation that can be estimated in the classic twin model. Following this, a systematic series of reduced nested models are fitted which test the significance of the A, and C/D parameters. A model just containing E is the null model (i.e. it is not possible to test the significance of E). The reduced models used are the AE model (dropping the C/D component), the CE/DE model (dropping the A component) and the E model (dropping both A and C/D components). To find the best fitting model, we adopt the rule of parsimony and aim to select the simplest model possible. We first look at the likelihood ratio comparison, which is chi-square distributed under the null, for the full univariate ACE/ADE models and their nested submodels – significance of this difference indicates whether dropping the parameter from the full model significantly worsens the model fit. When two or more submodels (e.g. DE and AE models) show that they both do not significantly worsen the fit of the model, we next look at the AIC values – submodels with a lower AIC suggest a better fit for the data^80^.

Sex differences in aetiology can be explored by looking at twin intra-class correlations for the different zygosity groups – MZ males, DZ males, MZ females, DZ females and DZ opposite sex pairs.

### Fitting the Bivariate Model

A bivariate Cholesky decomposition allows us to decompose the covariance between two traits, indicating the degree of genetic and environmental overlap between our measures. By way of example, we can look at the bivariate model of life satisfaction and support quality (Supplementary Figure S24). A first genetic factor represents genetic influences on life satisfaction. The extent to which these same genetic effects from this first genetic factor also influence support quality can then be estimated. A second genetic factor represents the genetic influence on support quality, which is independent of those shared with life satisfaction. The same decomposition is done for shared and non-shared environmental influences. We can then convert the results into the mathematically equivalent correlated factors solution (Supplementary Figure S25), which does not impose an order on the included variables and allows us to estimate genetic and environmental correlations between our measures^81^. We can also calculate the proportion of the phenotypic correlation between our measures of mental health and support that is accounted for by overlapping genetic or environmental influences.

### Data Availability

The dataset analysed during the current study is available on request from The Twins Early Development Study. Restrictions apply to the availability of these data, which were used under license for the current study, and so are not publicly available. Information about data access is available here: https://www.teds.ac.uk/research/collaborators-and-data/teds-data-access-policy. Data will be made available on request to interested researchers. As detailed on the above page, requests should be made via the data request form supplied. These requests will then be reviewed by the TEDS Executive committee.

## Electronic supplementary material

Supplementary Materials
