# Supplementary material for: Social support and mental health in late adolescence are correlated for genetic, as well as environmental, reasons
Source: Sci Rep. 2017 Oct 12;7:13088. doi: 10.1038/s41598-017-13449-2 (PMC5638798; doi:10.1038/s41598-017-13449-2)
Supplement: Supplementary file 1 — Supplementary Materials [file 41598_2017_13449_MOESM1_ESM.pdf]

Social support and mental health in late adolescence are correlated for genetic, as well as environmental, reasons

*R. Adele. H. Wang[1, 2, 3] * (BA), Oliver S. P. Davis[2, 3] (PhD), Robyn E. Wootton[1, 2, 3] (BSc), Abigail Mottershaw[1, 2, 3] (MSc), Claire M. A. Haworth[1, 2, 3] (PhD)*

[1] School of Experimental Psychology, University of Bristol, Bristol, United Kingdom

[2] MRC Integrative Epidemiology Unit, University of Bristol, Bristol, United Kingdom

[3] School of Social and Community Medicine, University of Bristol, Bristol, United Kingdom

*Corresponding author

Address: School of Experimental Psychology

University of Bristol

12a, Priory Road

Bristol BS8 1TU

United Kingdom

Telephone: +44 (0)117 3311078

Email: adele.wang@bristol.ac.uk

Supplementary Figures and Tables

Content

Supplementary Table S1. Twin Correlations across zygosity groups split by sex

Table showing within-trait cross-twin correlations obtained from the saturated univariate model through full information maximum likelihood across zygosity groups split by sex. These twin correlations were used to gauge the existence of qualitative and quantitative aetiological sex differences, both of which are suggested by our results

Supplementary Table S2. Means (Standard Deviation, SD) and ANOVA Results

Table showing means and standard deviations of the measures, unstandardized. Estimates were obtained from a subset consisting of one randomly selected twin from each twin pair, and were calculated for each measure across the whole sample, for monozygotic participants only, dizygotic participants only, males only and females only. Differences in measure scores between zygosity groups and between sex were investigated using ANOVA with eta-squared (proportion of variance attributed to an effect) used to look at the size of the effect of zygosity/sex on the measures.

Supplementary Table S3. Means and SD of individual traits across zygosity groups split by sex

Table showing means and standard deviations of the measures, unstandardized. Estimates were obtained from a subset consisting of one randomly selected twin from each twin pair, and were calculated for each measure within each zygosity pair split by sex.

Supplementary Table S4. Phenotypic correlations split across sex

Phenotypic correlations between social support measures and mental health measures for single sex male twins and single sex female twins to examine potential sex differences.

Supplementary Table 5. Fully saturated and constrained models to test twin assumptions

Table presenting the fit indices of each univariate saturated model and constrained submodel, obtained by structural equation model fitting, along with fit statistics comparing the fit of the constrained submodels (Sub1 and Sub2) to the fully saturated model (Sat). By testing for equality of variance across twin order and zygosity using our constrained submodels, we show that all our keep to the assumptions of equal variance.

Supplementary Table S6. Univariate Twin Analysis Results: Variance Components due to Additive Genetic (a^2), Non-Additive Genetic (d^2), Shared Environmental (c^2) and Non-Shared Environmental (e^2) effects.

Table presenting variance components due to additive genetic (a^2), non-additive genetic (d^2), shared environmental (c^2) and non-shared environmental (e^2) effects. Results were obtained from full univariate ACE/ADE models. ADE models are used when the DZ correlation is less than half of the MZ correlation. When DZ correlations are more than half of the MZ correlation, ACE models are used. Heritability estimates from these full models were similar to those from the reduced AE model (Table 2).

Supplementary Table S7. Univariate Twin Analysis Model Fit Statistics

Table presenting the fit statistics assessing the fit of each univariate model and submodel, obtained by structural equation model fitting. The best-fitting univariate model for each individual trait was selected based on AIC values, the fit of the model relative to the full/saturated model (as indicated by the likelihood ratio comparison) and parsimony.

Supplementary Table S8. Genetic and environmental correlations (95% confidence intervals)

Table showing estimates of genetic and environmental correlation between support and mental health measures. Estimates were obtained through bivariate AE model fitting. Most of the genetic correlations were moderate to high while the non-shared environmental correlations tended to be much lower.

Supplementary Table S9. Proportion of the phenotypic correlation explained by genetic and non-shared environmental overlaps (95% confidence intervals)

Table showing the proportion of the phenotypic correlation between our measures that is accounted for by common genetic or environmental influences. Estimates were obtained through bivariate AE model fitting. Genetic influences explained a larger proportion of the phenotypic correlations between all of the measures of mental health and support

Supplementary Table S10. Skew of measures

Table showing skew of measures. We transformed any measure which had a skew greater than 1 or less than -1.

Supplementary Figure S11: Comparing univariate results (with 95% confidence intervals) of transformed versus untransformed scales

For measures with an absolute skew value of over 1, we repeated our analyses on a reflected then log-transformed version of those scales with negative skew and directly log-transformed version of those scales with positive skew. This graph shows the a^2 and e^2 components obtained from our univariate analysis for the untransformed and transformed versions of the scales. Estimates were very similar, all with overlapping confidence intervals.

Supplementary Figure S12. Genetic correlations between transformed and untransformed measures of mental health and support quality (total) with 95% confidence intervals

Supplementary Figure S13. Genetic correlations between transformed and untransformed measures of mental health and support quality (significant other subscale) with 95% confidence intervals

Supplementary Figure S14. Genetic correlations between transformed and untransformed measures of mental health and support quality (family subscale) with 95% confidence intervals

Supplementary Figure S15. Genetic correlations between transformed and untransformed measures of mental health and support quality (friends subscale) with 95% confidence intervals

Supplementary Figures S13-16 show only genetic correlations between support quality and mental health. Each bar represents the genetic correlation between a mental health measure and support quality (total score/significant other subscale/family subscale/friend subscale). The support quality total score and subscales all had a negative skew less than -1 meaning that we reflected and then log-transformed the scale/subscales. We then obtained the genetic correlation between the untransformed and transformed versions of these (sub)scales with untransformed and (where absolute skew was greater than 1) transformed versions of individual mental health measures. Estimates were very similar for untransformed and transformed measures, with overlapping confidence intervals.

Supplementary Figure S16. Genetic correlations between transformed and untransformed measures of mental health and support quantity with 95% confidence intervals

Figure showing only genetic correlations between support quantity and mental health. Support quantity had a skew of between 0 and -1 so was not transformed. This untransformed score was correlated with untransformed and transformed versions of mental health measures which had an absolute skew value

greater than 1. For those mental health measures with an absolute skew value of less than 1, results are already presented in Figure 1. Estimates were very similar for untransformed and transformed measures, with overlapping confidence intervals.

Supplementary Figure S17. Environmental correlations between transformed and untransformed measures of mental health and support quality (total) with 95% confidence intervals

Supplementary Figure S18. Environmental correlations between transformed and untransformed measures of mental health and support quality (significant other) with 95% confidence intervals

Supplementary Figure S19. Environmental correlations between transformed and untransformed measures of mental health and support quality (family) with 95% confidence intervals

Supplementary Figure S20. Environmental correlations between transformed and untransformed measures of mental health and support quality (friends) with 95% confidence intervals

Supplementary Figure S21. Environmental correlations between transformed and untransformed measures of mental health and support quantity with 95% confidence intervals

Supplementary Figures S18-S22 parallel the results presented in Supplementary Figures S13-S16 except here these figures present the environmental correlations between untransformed and transformed support and mental health measures. Again results were similar with overlapping confidence intervals.

Supplementary Figure S22: Illustration of the basic twin model for one trait of one twin (within a twin pair)

Figure illustrating the basic twin model path diagram which is used to estimate genetic and environmental influences which contribute to the phenotypic variance of a trait.

Supplementary Figure S23. A path diagram of the basic twin model

Figure showing the genetic and environmental relationships within twins for one trait and forms the basis of all twin analyses. The total observed phenotypic variance of a trait is the sum of additive genetic (A), shared environmental (C), and non-shared environmental (E) influences, shown as latent factors.

Supplementary Figure S24: Illustration of the Cholesky Decomposition

Figuring showing the bivariate Cholesky decomposition which allows us to decompose the covariance between two traits, indicating the degree of genetic and environmental overlap between our measures.

Supplementary Figure S25: Illustration of the Correlated Factors Solution

Figure showing the correlated factors solution, which is mathematically equivalent to the bivariate Cholesky decomposition. It does not impose an order on the included variables and allows us to estimate genetic and environmental correlations between our measures. We can also calculate the proportion of the phenotypic correlation between our measures of mental health and support that is accounted for by overlapping genetic or environmental influences.

Supplementary Table S1. Twin Correlations across zygosity groups split by sex

	MZ all	DZ all	MZ males	DZ males	MZ females	DZ females	DZ same sex	DZ opposite sex
Positive affect	0.49	0.19	0.54	0.30	0.47	0.32	0.31	0.08
	(0.41, 0.57)	(0.12, 0.26)	(0.39, 0.67)	(0.13, 0.45)	(0.37, 0.56)	(0.21, 0.42)	(0.22, 0.40)	(-0.02, 0.18)
Negative affect	0.38	0.15	0.38	-0.00	0.39	0.21	0.17	0.12
	(0.29, 0.47)	(0.08, 0.21)	(0.20, 0.54)	(-0.18, 0.18)	(0.28, 0.48)	(0.10, 0.32)	(0.07, 0.26)	(0.02, 0.22)
Subjective happiness	0.47	0.16	0.46	0.04	0.48	0.30	0.23	0.10
	(0.39, 0.55)	(0.10, 0.23)	(0.29, 0.60)	(-0.14, 0.22)	(0.37, 0.56)	(0.18, 0.40)	(0.14, 0.32)	(-0.00, 0.20)
Life satisfaction	0.57	0.34	0.42	0.32	0.60	0.46	0.43	0.25
	(0.5, 0.64)	(0.28, 0.40)	(0.25, 0.57)	(0.15, 0.47)	(0.52, 0.68)	(0.37, 0.55)	(0.35, 0.51)	(0.15, 0.34)
Gratitude	0.58	0.22	0.46	0.25	0.61	0.33	0.31	0.13
	(0.51, 0.65)	(0.15, 0.29)	(0.29, 0.60)	(0.07, 0.41)	(0.53, 0.68)	(0.22, 0.43)	(0.21, 0.39)	(0.03, 0.23)
Meaning in life	0.53	0.23	0.68	0.31	0.48	0.33	0.33	0.14
	(0.45, 0.60)	(0.16, 0.29)	(0.56, 0.77)	(0.14, 0.46)	(0.38, 0.57)	(0.22, 0.43)	(0.24, 0.41)	(0.04, 0.23)
Autonomy	0.52	0.23	0.48	0.17	0.53	0.34	0.30	0.15
	(0.44, 0.60)	(0.16, 0.29)	(0.31, 0.62)	(-0.01, 0.34)	(0.44, 0.62)	(0.24, 0.44)	(0.21, 0.39)	(0.05, 0.25)
Competence	0.51	0.23	0.53	0.24	0.49	0.36	0.33	0.12
	(0.42, 0.58)	(0.16, 0.30)	(0.37, 0.66)	(0.06, 0.40)	(0.39, 0.58)	(0.25, 0.45)	(0.24, 0.41)	(0.02, 0.22)
Relatedness	0.57	0.21	0.55	0.26	0.58	0.35	0.32	0.10
	(0.50, 0.64)	(0.14, 0.28)	(0.40, 0.68)	(0.09, 0.42)	(0.49, 0.65)	(0.24, 0.45)	(0.23, 0.41)	(-0.00, 0.20)
Depression	0.41	0.20	0.45	0.10	0.41	0.33	0.29	0.08
	(0.32, 0.50)	(0.13, 0.26)	(0.28, 0.59)	(-0.10, 0.29)	(0.30, 0.50)	(0.22, 0.43)	(0.20, 0.38)	(-0.02, 0.18)
Support quantity	0.49	0.27	0.48	0.29	0.50	0.36	0.34	0.21
	(0.41, 0.57)	(0.21, 0.34)	(0.31, 0.62)	(0.11, 0.44)	(0.40, 0.58)	(0.26, 0.46)	(0.25, 0.43)	(0.11, 0.30)
Support Quality (*total)*	0.55	0.27	0.47	0.33	0.57	0.32	0.32	0.22

	(0.48, 0.62)	(0.2, 0.33)	(0.30, 0.61)	(0.16, 0.48)	(0.48, 0.65)	(0.21, 0.42)	(0.23, 0.41)	(0.12, 0.31)
Support Quality	0.44	0.21	0.48	0.26	0.42	0.26	0.25	0.17
(*significant other)*	(0.35, 0.52)	(0.15, 0.28)	(0.32, 0.62)	(0.08, 0.42)	(0.32, 0.52)	(0.14, 0.36)	(0.16, 0.34)	(0.07, 0.26)
	0.59	0.31	0.41	0.28	0.63	0.36	0.34	0.28
Support Quality (*family)*	(0.52, 0.65)	(0.25, 0.37)	(0.23, 0.56)	(0.11, 0.44)	(0.55, 0.70)	(0.25, 0.45)	(0.25, 0.42)	(0.19, 0.37)
	0.40	0.15	0.34	0.23	0.42	0.24	0.23	0.07
Support Quality (*friends)*	(0.31, 0.49)	(0.08, 0.22)	(0.15, 0.50)	(0.05, 0.39)	(0.31, 0.51)	(0.12, 0.34)	(0.14, 0.33)	(-0.03, 0.17)

Note Twin correlations were obtained through maximum likelihood from the constrained saturated univariate model. We used within trait cross twin correlations from our saturated model, which constrained means across sex and age. Twin pairs: total Npairs = 1,158, $N_{MZM\ pairs}$ = 101, $N_{DZM\ pairs}$ = 122, $N_{MZF\ pairs}$ = 259, $N_{DZF\ pairs}$ = 282, $N_{DZO\ pairs}$ = 394 Observing the patterns of twin correlations between MZ male and DZ males compared to MZ females and DZ females, we find evidence of potential quantitative sex differences, where the same genetic and environmental factors influence both males and females but to varying extents. DZ opposite sex twin correlations are generally lower than DZ same sex twin correlations. Therefore, it seems like there may be qualitative sex differences, where there are different genetic and environmental influences on a trait for females and males. However, many of the confidence intervals overlap, and equally, we cannot make any inferences from our point estimates due to our small sample sizes. The small sample sizes within each zygosity group in this study means that we are underpowered to run a sex-limitation model.

Supplementary Table S2. Means (Standard Deviation, SD) and ANOVA Results

	All N=1141-1144	MZ N=356-357	DZ N=784-787	Male N=417-418	Female N=724-726	Zygosity p-value	Zygosity effect size	Sex p-value	Sex effect size
Positive Affect	33.74 (7.61)	33.77 (7.32)	33.73 (7.74)	34.62 (7.48)	33.24 (7.64)	0.94	5.64E-06	3.20E-03**	7.59E-03
Negative Affect	18.87 (6.74)	18.77 (6.47)	18.92 (6.86)	17.39 (6.19)	19.73 (6.89)	0.72	1.16E-04	1.12E-08***	2.82E-02
Subjective Happiness	5.16 (1.02)	5.22 (0.94)	5.13 (1.05)	5.20 (0.99)	5.14 (1.03)	0.17	1.62E-03	0.30	9.32E-04
Life Satisfaction	5.67 (1.01)	5.73 (0.97)	5.65 (1.02)	5.76 (0.94)	5.63 (1.04)	0.21	1.38E-03	0.03*	3.91E-03
Gratitude	5.81 (0.90)	5.86 (0.89)	5.79 (0.90)	5.73 (0.84)	5.86 (0.93)	0.25	1.14E-03	0.01*	5.25E-03
Meaning in Life	5.12 (1.18)	5.15 (1.16)	5.10 (1.18)	5.17 (1.12)	5.09 (1.21)	0.48	4.39E-04	0.29	9.62E-04
Autonomy	5.04 (0.85)	5.08 (0.85)	5.03 (0.85)	5.02 (0.82)	5.06 (0.87)	0.39	6.40E-04	0.45	5.06E-04
Competence	4.98 (1.01)	5.00 (1.01)	4.97 (1.02)	5.01 (0.95)	4.96 (1.05)	0.59	2.53E-04	0.39	6.58E-04
Relatedness	5.76 (0.84)	5.79 (0.83)	5.75 (0.85)	5.67 (0.83)	5.81 (0.84)	0.45	4.94E-04	7.75E-03**	6.21E-03
Depression	9.76 (5.56)	9.76 (5.66)	9.76 (5.52)	9.06 (4.60)	10.16 (6.01)	1.00	2.28E-09	1.29E-03**	9.05E-03
Support Quantity	15.23 (4.53)	14.88 (4.59)	15.38 (4.50)	14.92 (4.49)	15.40 (4.55)	0.08	2.64E-03	8.58E-02	2.59E-03
Support Quality (*total)*	5.64 (1.00)	5.71 (0.96)	5.61 (1.01)	5.53 (0.96)	5.71 (1.01)	0.13	1.97E-03	2.85E-03**	7.77E-03
Support Quality (*significant other)*	5.65 (1.29)	5.77 (1.22)	5.60 (1.32)	5.39 (1.27)	5.80 (1.28)	0.04*	3.56E-03	2.20E-07***	2.32E-02
Support Quality (*family)*	5.69 (1.14)	5.73 (1.18)	5.68 (1.13)	5.71 (1.05)	5.68 (1.19)	0.43	5.52E-04	7.02E-01	1.28E-04
Support Quality (*friends)*	5.58 (1.17)	5.62 (1.14)	5.56 (1.19)	5.48 (1.10)	5.64 (1.21)	0.42	5.58E-04	0.02*	4.74E-03

Note. Table showing means and standard deviations of the measures, unstandardized. Estimates were obtained from a subset consisting of one randomly selected twin from each twin pair, and were calculated for each measure across the whole sample, for monozygotic participants only, dizygotic participants only, males only and females only. Differences in measure scores between zygosity groups and between sex were investigated using ANOVA with eta-squared (proportion of variance attributed to an effect) used to look at the size of the effect of zygosity/sex on the measures.

*p<0.05, **p<0.01, ***p<0.001. MZ= monozygotic twins; DZ = dizygostic twins (same and opposite sex)

Supplementary Table S3. Means and SD of individual traits across zygosity groups split by sex

Measure	MZ males	DZ males	MZ females	DZ females	DZ opposite sex	ANOVA p value	ANOVA effect size
Positive affect	35.39 (7.10)<br>N= 100	34.66 (8.04)<br>N= 119	33.14 (7.33)<br>N= 257	33.38 (7.30)<br>N= 280	33.70 (7.95)<br>N= 387	8.28E-02	2.63E-03
Negative affect	17.19 (6.03)<br>N= 100	16.75 (5.80)<br>N= 119	19.38 (6.54)<br>N= 257	19.58 (6.65)<br>N= 280	19.12 (7.19)<br>N= 387	8.23E-04***	9.76E-03
Subjective happiness	5.33 (0.81)<br>N= 99	5.29 (1.03)<br>N= 119	5.18 (0.98)<br>N= 257	5.13 (1.03)<br>N= 280	5.09 (1.07)<br>N= 386	8.54E-03**	6.06E-03
Life satisfaction	5.93 (0.82)<br>N= 100	5.83 (0.92)<br>N= 119	5.65 (1.01)<br>N= 257	5.58 (1.04)<br>N= 280	5.64 (1.04)<br>N= 386	4.56E-03**	7.04E-03
Gratitude	5.79 (0.80)<br>N= 100	5.85 (0.83)<br>N= 119	5.88 (0.93)<br>N= 257	5.87 (0.86)<br>N= 280	5.71 (0.95)<br>N= 387	0.13	2.02E-03
Meaning in life	5.32 (1.09)<br>N= 100	5.28 (1.14)<br>N= 119	5.09 (1.19)<br>N= 256	5.15 (1.10)<br>N= 280	5.01 (1.25)<br>N= 387	8.75E-03**	6.01E-03
Autonomy	5.12 (0.79)<br>N= 100	5.11 (0.77)<br>N= 118	5.06 (0.88)<br>N= 257	5.07 (0.86)<br>N= 279	4.98 (0.86)<br>N= 387	6.58E-02	2.97E-03
Competence	5.19 (0.95)<br>N= 100	5.04 (0.97)<br>N= 118	4.93 (1.02)<br>N= 257	4.99 (1.05)<br>N= 281	4.93 (1.01)<br>N= 387	5.73E-02	3.16E-03
Relatedness	5.71 (0.82)<br>N= 100	5.79 (0.79)<br>N= 118	5.82 (0.83)<br>N= 257	5.81 (0.80)<br>N= 279	5.69 (0.89)<br>N= 387	0.34	7.97E-04
Depression	8.66 (4.21)<br>N= 100	8.43 (4.19)<br>N= 119	10.19 (6.09)<br>N= 256	9.94 (5.70)<br>N= 280	10.03 (5.69)<br>N= 387	7.68E-03**	6.22E-03
Support Quantity	14.93 (4.33)<br>N= 100	15.35 (4.63)<br>N= 119	14.86 (4.70)<br>N= 257	15.52 (4.46)<br>N= 281	15.29 (4.49)<br>N= 386	0.36	7.29E-04

Support Quality (*total*)	5.63 (0.86)	5.53 (1.03)	5.74 (1.00)	5.66 (1.00)	5.61 (1.02)	0.90	1.34E-05
	N= 100	N= 119	N= 257	N= 281	N= 387		
Support Quality (*significant other*)	5.41 (1.16)	5.41 (1.32)	5.90 (1.22)	5.73 (1.27)	5.56 (1.34)	0.60	2.37E-04
	N= 100	N= 119	N= 257	N= 281	N= 387		
Support Quality (*family*)	5.91 (0.92)	5.68 (1.14)	5.67 (1.26)	5.65 (1.17)	5.69 (1.09)	0.28	1.02E-03
	N= 100	N= 119	N= 257	N= 281	N= 387		
Support Quality (*friends*)	5.56 (0.98)	5.48 (1.16)	5.65 (1.20)	5.58 (1.21)	5.57 (1.18)	0.84	3.61E-05
	N= 100	N= 119	N= 257	N= 281	N= 387		

Note. Table showing means and standard deviations of the measures, unstandardized. Estimates were obtained from a subset consisting of one randomly selected twin from each twin pair, and were calculated for each measure within each zygosity pair split by sex. Differences in measure scores between zygosity groups were investigated using ANOVA with eta-squared (proportion of variance attributed to an effect) used to look at the size of the effect of zygosity/sex on the measures.

*p<0.05, **p<0.01, ***p<0.001. MZ= monozygotic twins; DZ = dizygotic twins

Supplementary Table S4. Phenotypic correlations split across sex

	Support Quantity		Support Quality (*total*)	
	**Males**	**Females**	**Males**	**Females**
Positive affect	0.36 (0.27, 0.44)	0.36 (0.31, 0.42)	0.39 (0.31, 0.48)	0.47 (0.42, 0.52)
Negative affect	-0.15 (-0.24, -0.05)	-0.19 (-0.25, -0.13)	-0.21 (-0.30, -0.12)	-0.35 (-0.41, -0.29)
Subjective happiness	0.38 (0.30, 0.46)	0.34 (0.28, 0.40)	0.46 (0.38, 0.54)	0.57 (0.53, 0.61)
Life satisfaction	0.39 (0.31, 0.47)	0.45 (0.39, 0.50)	0.66 (0.60, 0.72)	0.70 (0.67, 0.73)
Gratitude	0.31 (0.22, 0.40)	0.38 (0.32, 0.44)	0.52 (0.44, 0.59)	0.60 (0.56, 0.64)
Meaning in life	0.32 (0.22, 0.41)	0.32 (0.26, 0.37)	0.53 (0.46, 0.60)	0.54 (0.50, 0.59)
Autonomy	0.36 (0.27, 0.45)	0.36 (0.30, 0.41)	0.47 (0.38, 0.54)	0.54 (0.50, 0.59)
Competence	0.33 (0.24, 0.42)	0.34 (0.28, 0.40)	0.47 (0.39, 0.55)	0.52 (0.47, 0.56)
Relatedness	0.64 (0.57, 0.70)	0.61 (0.57, 0.65)	0.64 (0.57, 0.69)	0.66 (0.63, 0.70)
Depression	-0.29 (-0.38, -0.20)	-0.32 (-0.37, -0.26)	-0.42 (-0.50, -0.33)	-0.53 (-0.57, -0.48)

Note. Phenotypic correlations between social support measures and mental health measures for same sex male twins and same sex female twins. Phenotypic correlations were obtained using MZ and DZ single sex twins through full information maximum likelihood by running a fully saturated bivariate model and then constraining the model so that the cross-twin cross-trait correlations equate across twin order and zygosity. With regards to support quantity, its correlations with mental health are very similar across males and females. With regards to support quality, the trend suggests that its association with mental health is higher for females, however confidence intervals overlap.

Supplementary Table S5. Fully saturated and constrained models to test twin assumptions

Measure	Model	Model Fit					
		**-2LL**	**df**	**AIC**	**$\Delta\chi^2$**	**Δ df**	**p**
	Sat	6275.02	2253	1769.02	NA	NA	NA
Positive Affect	Sub1	6276.97	2255	1766.97	1.95	2	0.38
	Sub2	6277.33	2256	1765.33	2.31	3	0.51
	Sat	6261.22	2253	1755.22	NA	NA	NA
Negative Affect	Sub1	6270.19	2255	1760.19	8.97	2	0.01
	Sub2	6272.14	2256	1760.14	10.92	3	0.01
	Sat	6280.45	2251	1778.45	NA	NA	NA
Subjective Happiness	Sub1	6289.95	2253	1783.95	9.50	2	8.64E-03
	Sub2	6290.33	2254	1782.33	9.88	3	0.02
	Sat	6158.89	2252	1654.89	NA	NA	NA
Life Satisfaction	Sub1	6159.32	2254	1651.32	0.44	2	0.80
	Sub2	6160.46	2255	1650.46	1.58	3	0.66
	Sat	6210.02	2254	1702.02	NA	NA	NA
Gratitude	Sub1	6210.03	2256	1698.03	0.01	2	0.99
	Sub2	6210.03	2257	1696.03	0.01	3	1.00
	Sat	6251.66	2252	1747.66	NA	NA	NA
Meaning in Life	Sub1	6252.01	2254	1744.01	0.34	2	0.84
	Sub2	6252.78	2255	1742.78	1.11	3	0.77
	Sat	6236.42	2252	1732.42	NA	NA	NA
Autonomy	Sub1	6237.16	2254	1729.16	0.74	2	0.69
	Sub2	6237.18	2255	1727.18	0.76	3	0.86
Competence	Sat	6260.62	2254	1752.62	NA	NA	NA

	Sub1	6260.64	2256	1748.64	0.02	2	0.99
	Sub2	6260.70	2257	1746.70	0.08	3	0.99
Relatedness	Sat	6220.11	2251	1718.11	NA	NA	NA
	Sub1	6221.20	2253	1715.20	1.09	2	0.58
	Sub2	6221.24	2254	1713.24	1.13	3	0.77
Depression	Sat	6283.27	2252	1779.27	NA	NA	NA
	Sub1	6283.46	2254	1775.46	0.19	2	0.91
	Sub2	6283.85	2255	1773.85	0.58	3	0.90
Support quantity	Sat	6250.42	2251	1748.42	NA	NA	NA
	Sub1	6251.12	2253	1745.12	0.71	2	0.70
	Sub2	6253.34	2254	1745.34	2.92	3	0.40
Support quality (*total*)	Sat	6191.85	2254	1683.85	NA	NA	NA
	Sub1	6196.58	2256	1684.58	4.73	2	0.09
	Sub2	6196.67	2257	1682.67	4.82	3	0.19
Support quality (*significant other*)	Sat	6234.68	2254	1726.68	NA	NA	NA
	Sub1	6242.89	2256	1730.89	8.21	2	0.02
	Sub2	6242.90	2257	1728.90	8.22	3	0.04
Support quality (*family*)	Sat	6172.86	2254	1664.86	NA	NA	NA
	Sub1	6173.30	2256	1661.30	0.44	2	0.80
	Sub2	6174.15	2257	1660.15	1.29	3	0.73
Support quality (*friend*)	Sat	6317.42	2255	1807.42	NA	NA	NA
	Sub1	6320.73	2257	1806.73	3.31	2	0.19
	Sub2	6321.04	2258	1805.04	3.62	3	0.31

Note. Table presenting the fit indices of each univariate saturated model and constrained submodel, obtained by structural equation model fitting, along with fit statistics comparing the fit of the constrained submodels (Sub1 and Sub2) to the fully saturated model (Sat). Our saturated model was constructed from MZs and DZs combined

across sex. In our saturated model, we account for the effects of covariates (age and sex) from our traits using a Means Model. This model specifies one Means Model across twins and zygosity group. The saturated model includes a covariance within twin pairs for each zygosity (between MZ twin 1 and MZ twin 2 and between DZ twin 1 and DZ twin 2), and variances for each person-category (MZ twin 1, MZ twin 2, DZ twin 1, DZ twin 2). The constrained submodels allows us to check the assumptions of the twin method. We can test equality of variance across twin order within zygosity (sub1) and equality of variance across twin order and across zygosity (sub2)

−2LL = negative 2 log likelihood; df = degrees of freedom; AIC = Akaike's Information Criterion; Δχ2 = difference in chi square; Δdf = difference in degrees of freedom; p = p-value. Bonferroni corrected alpha level, correcting for multiple testing (30 tests) = 0.001667. -2LL provides a relative measure of model fit as differences in -2LL between models are distributed as χ2. Therefore the significance of Δχ2 provides an indication of the constrained sub model being a significantly worse fit to the data than the fully saturated model. Lower AIC values reflect better model fit.

Equating variances across twin pair and zygosity did not significantly worsen fit for any of our measures, using a Bonferroni corrected p threshold (lowered for multiple testing).

Supplementary Table S6. Univariate Twin Analysis Results: Variance Components due to Additive Genetic (a^2), Non-Additive Genetic (d^2), Shared Environmental (c^2) and Non-Shared Environmental (e^2) effects.

	a^2	c^2	d^2	e^2
Positive Affect	0.25 (0.00, 0.51)		0.25 (0.00, 0.54)	0.50 (0.43, 0.58)
Negative Affect	0.23 (0.00, 0.41)		0.13 (0.00, 0.43)	0.64 (0.56, 0.72)
Subjective Happiness	0.17 (0.00, 0.45)		0.31 (0.00, 0.54)	0.52 (0.45, 0.60)
Life Satisfaction	0.51 (0.34, 0.64)	0.08 (0.00, 0.21)		0.41 (0.35, 0.48)
Gratitude	0.31 (0.03, 0.57)		0.27 (0.00, 0.57)	0.42 (0.36, 0.49)
Meaning in Life	0.36 (0.08, 0.57)		0.18 (0.00, 0.47)	0.46 (0.40, 0.53)
Autonomy	0.39 (0.11, 0.57)		0.14 (0.00, 0.44)	0.47 (0.41, 0.55)
Competence	0.41 (0.14, 0.56)		0.10 (0.00, 0.39)	0.49 (0.42, 0.57)
Relatedness	0.27 (0.00, 0.54)		0.29 (0.00, 0.59)	0.43 (0.37, 0.50)
Depression	0.39 (0.10, 0.47)		0.02 (0.00, 0.34)	0.60 (0.52, 0.67)
Support Quantity	0.38 (0.19, 0.54)	0.09 (0.00, 0.23)		0.53 (0.46, 0.60)
Support Quality (*total)*	0.53 (0.26, 0.60)		0.02 (0.00, 0.31)	0.45 (0.39, 0.52)
Support Quality (*significant other)*	0.42 (0.14, 0.50)		0.02 (0.00, 0.33)	0.56 (0.49, 0.64)
Support Quality (*family)*	0.52 (0.35, 0.63)	0.05 (0.00, 0.19)		0.42 (0.37, 0.49)
Support Quality (*friends)*	0.22 (0.00, 0.43)		0.18 (0.00, 0.46)	0.60 (0.53, 0.69)

Note. Table presenting variance components due to additive genetic (a^2), non-additive genetic (d^2), shared environmental (c^2) and non-shared environmental (e^2) effects. Results were obtained from full univariate ACE/ADE models. ADE models are used when the DZ correlation is less than half of the MZ correlation. When DZ correlations are more than half of the MZ correlation, ACE models are used. Heritability estimates from these full models were similar to those from the reduced AE model (Table 2).

Supplementary Table S7. Univariate Twin Analysis Model Fit Statistics

Measure	Model	Model Fit					
		-2LL	df	AIC	$\Delta\chi^2$	Δ df	p
Positive Affect	Sat	6275.02	2253	1769.02	-	-	-
	ADE*	6277.33	2256	1765.33	2.31	3	0.51
	DE	6280.50	2257	1766.50	3.17	1	7.49E-02
	AE	6280.02	2257	1766.02	2.69	1	0.10
Negative Affect	Sat	6261.22	2253	1755.22	-	-	-
	ADE*	6272.14	2256	1760.14	10.92	3	1.22E-02
	DE	6274.56	2257	1760.56	2.42	1	0.12
	AE	6272.75	2257	1758.75	0.61	1	0.43
Subjective Happiness	Sat	6280.45	2251	1778.45	-	-	-
	ADE*	6290.33	2254	1782.33	9.88	3	1.96E-02
	DE	6291.77	2255	1781.77	1.44	1	0.23
	AE	6294.22	2255	1784.22	3.89	1	4.85E-02
Life Satisfaction	Sat	6158.89	2252	1654.89	-	-	-
	ACE*	6160.46	2255	1650.46	1.58	3	0.66
	CE	6189.81	2256	1677.81	29.35	1	6.03E-08
	AE	6161.78	2256	1649.78	1.32	1	0.25
Gratitude	Sat	6210.02	2254	1702.02	-	-	-
	ADE*	6210.03	2257	1696.03	0.01	3	1.00
	DE	6214.81	2258	1698.81	4.78	1	2.88E-02
	AE	6213.58	2258	1697.58	3.55	1	5.96E-02
Meaning in Life	Sat	6251.66	2252	1747.66	-	-	-
	ADE*	6252.78	2255	1742.78	1.11	3	0.77

	DE	6259.52	2256	1747.52	6.74	1	9.41E-03
	AE	6254.28	2256	1742.28	1.50	1	0.22
Autonomy	Sat	6236.42	2252	1732.42	-	-	-
	ADE*	6237.18	2255	1727.18	0.76	3	0.86
	DE	6244.60	2256	1732.60	7.42	1	6.46E-03
	AE	6238.08	2256	1726.08	0.90	1	0.34
Competence	Sat	6260.62	2254	1752.62	-	-	-
	ADE*	6260.70	2257	1746.70	0.08	3	0.99
	DE	6269.21	2258	1753.21	8.51	1	3.54E-03
	AE	6261.12	2258	1745.12	0.43	1	0.51
Relatedness	Sat	6220.11	2251	1718.11	-	-	-
	ADE*	6221.24	2254	1713.24	1.13	3	0.77
	DE	6224.96	2255	1714.96	3.72	1	5.39E-02
	AE	6225.22	2255	1715.22	3.98	1	4.61E-02
Depression	Sat	6283.27	2252	1779.27	-	-	-
	ADE*	6283.85	2255	1773.85	0.58	3	0.90
	DE	6290.70	2256	1778.70	6.85	1	8.88E-03
	AE	6283.87	2256	1771.87	0.01	1	0.91
Support Quantity	Sat	6250.42	2251	1748.42	-	-	-
	ACE*	6253.34	2254	1745.34	2.92	3	0.40
	CE	6267.61	2255	1757.61	14.27	1	1.58E-04
	AE	6254.69	2255	1744.69	1.36	1	0.24
Support Quality (*total*)	Sat	6191.85	2254	1683.85	-	-	-
	ADE*	6196.67	2257	1682.67	4.82	3	0.19
	DE	6210.89	2258	1694.89	14.21	1	1.63E-04

	AE	6196.69	2258	1680.69	0.01	1	0.91
Support Quality	Sat	6234.68	2254	1726.68	-	-	-
(*significant other*)	ADE*	6242.90	2257	1728.90	8.22	3	4.18E-02
	DE	6251.27	2258	1735.27	8.37	1	3.81E-03
	AE	6242.91	2258	1726.91	0.02	1	0.90
Support Quality	Sat	6172.86	2254	1664.86	-	-	-
(*family*)	ACE*	6174.15	2257	1660.15	1.29	3	0.73
	CE	6205.23	2258	1689.23	31.08	1	2.47E-08
	AE	6174.75	2258	1658.75	0.60	1	0.44
Support Quality	Sat	6317.42	2255	1807.42	-	-	-
(*friend*)	ADE*	6321.04	2258	1805.04	3.62	3	0.31
	DE	6323.16	2259	1805.16	2.12	1	0.15
	AE	6322.27	2259	1804.27	1.24	1	0.27

Note. Table presenting the fit statistics assessing the fit of each univariate model and submodel, obtained by structural equation model fitting. The best-fitting univariate model for each individual trait was selected based on AIC values, the fit of the model relative to the full/saturated model (as indicated by the likelihood ratio comparison) and parsimony.

−2LL = negative 2 log likelihood; df = degrees of freedom; AIC = Akaike's Information Criterion; Δχ2 = difference in chi square; Δdf = difference in degrees of freedom; p = p-value. Bonferroni corrected alpha level, correcting for multiple testing (15 tests) = 0.0033. -2LL provides a relative measure of model fit as differences in -2LL between models are distributed as χ2. Therefore the significance of Δχ2 provides an indication of the sub model being a significantly worse fit to the data than the full model. Lower AIC values reflect better model fit.

Sat = saturated model with means and variances constrained across sex and age. The saturated model describes the data with the maximum number of free parameters. The saturated model includes a covariance within twin pairs for each zygosity (between MZ twin 1 and MZ twin 2 and between DZ twin 1 and DZ twin 2), and variances and means for each person-category (MZ twin 1, MZ twin 2, DZ twin 1, DZ twin 2). Goodness of fit of the ACE/ADE models are compared relative to the perfectly fitting saturated model. *Fit of nested models DE/CE and AE given relative to full ADE/ACE model

Supplementary Table S8. Genetic and environmental correlations (95% confidence intervals)

		Genetic Correlation	Unique Environmental Correlation
Positive Affect	Support Quantity	0.56 (0.47, 0.65)	0.20 (0.12, 0.28)
	Support Quality (*total)*	0.64 (0.55, 0.73)	0.24 (0.15, 0.32)
	Support Quality (*significant other)*	0.62 (0.51, 0.73)	0.15 (0.06, 0.23)
	Support Quality (*family)*	0.51 (0.42, 0.60)	0.22 (0.13, 0.30)
	Support Quality (*friends)*	0.59 (0.48, 0.71)	0.19 (0.10, 0.27)
Negative Affect	Support Quantity	-0.29 (-0.41, -0.16)	-0.13 (-0.21, -0.04)
	Support Quality (*total)*	-0.54 (-0.66, -0.43)	-0.15 (-0.24, -0.07)
	Support Quality (*significant other)*	-0.39 (-0.53, -0.24)	-0.07 (-0.16, 0.01)
	Support Quality (*family)*	-0.53 (-0.64, -0.42)	-0.18 (-0.26, -0.09)
	Support Quality (*friends)*	-0.54 (-0.69, -0.40)	-0.12 (-0.20, -0.04)
Subjective Happiness	Support Quantity	0.61 (0.51, 0.70)	0.15 (0.07, 0.23)
	Support Quality (*total)*	0.81 (0.73, 0.89)	0.31 (0.23, 0.38)
	Support Quality (*significant other)*	0.67 (0.57, 0.78)	0.22 (0.13, 0.30)
	Support Quality (*family)*	0.72 (0.64, 0.80)	0.27 (0.19, 0.35)
	Support Quality (*friends)*	0.79 (0.68, 0.91)	0.22 (0.14, 0.30)
Life satisfaction	Support Quantity	0.61 (0.53, 0.69)	0.22 (0.14, 0.30)
	Support Quality (*total)*	0.82 (0.77, 0.87)	0.50 (0.43, 0.57)
	Support Quality (*significant other)*	0.66 (0.58, 0.75)	0.29 (0.21, 0.37)
	Support Quality (*family)*	0.74 (0.68, 0.79)	0.47 (0.40, 0.54)

	Support Quality (*friends*)	0.80 (0.73, 0.88)	0.42 (0.35, 0.49)
Gratitude	Support Quantity	0.53 (0.44, 0.62)	0.18 (0.09, 0.26)
	Support Quality (*total)*	0.80 (0.73, 0.86)	0.32 (0.24, 0.40)
	Support Quality (*significant other)*	0.69 (0.60, 0.77)	0.22 (0.14, 0.30)
	Support Quality (*family)*	0.77 (0.71, 0.84)	0.29 (0.21, 0.37)
	Support Quality (*friends*)	0.67 (0.57, 0.76)	0.23 (0.14, 0.31)
Meaning in Life	Support Quantity	0.56 (0.46, 0.65)	0.10 (0.02, 0.19)
	Support Quality (*total)*	0.77 (0.70, 0.84)	0.28 (0.20, 0.36)
	Support Quality (*significant other)*	0.73 (0.64, 0.81)	0.17 (0.09, 0.25)
	Support Quality (*family)*	0.66 (0.58, 0.73)	0.27 (0.18, 0.35)
	Support Quality (*friends*)	0.68 (0.57, 0.78)	0.21 (0.13, 0.29)
Autonomy	Support Quantity	0.49 (0.40, 0.58)	0.25 (0.17, 0.33)
	Support Quality (*total)*	0.72 (0.64, 0.79)	0.35 (0.27, 0.42)
	Support Quality (*significant other)*	0.62 (0.52, 0.72)	0.17 (0.09, 0.25)
	Support Quality (*family)*	0.70 (0.62, 0.77)	0.28 (0.20, 0.36)
	Support Quality (*friends*)	0.59 (0.49, 0.69)	0.35 (0.27, 0.43)
Competence	Support Quantity	0.51 (0.42, 0.60)	0.22 (0.13, 0.30)
	Support Quality (*total)*	0.77 (0.70, 0.84)	0.26 (0.18, 0.34)
	Support Quality (*significant other)*	0.69 (0.59, 0.79)	0.15 (0.06, 0.23)
	Support Quality (*family)*	0.70 (0.63, 0.78)	0.22 (0.14, 0.30)
	Support Quality (*friends*)	0.67 (0.56, 0.77)	0.23 (0.15, 0.31)

Relatedness	Support Quantity	0.72 (0.66, 0.79)	0.49 (0.42, 0.55)
	Support Quality (*total)*	0.85 (0.79, 0.90)	0.44 (0.37, 0.51)
	Support Quality (*significant other)*	0.74 (0.65, 0.83)	0.24 (0.16, 0.32)
	Support Quality (*family)*	0.71 (0.64, 0.79)	0.22 (0.14, 0.30)
	Support Quality (*friends)*	0.83 (0.76, 0.90)	0.53 (0.46, 0.59)
Depression	Support Quantity	-0.54 (-0.65, -0.44)	-0.14 (-0.22, -0.06)
	Support Quality (*total)*	-0.74 (-0.82, -0.65)	-0.31 (-0.38, -0.23)
	Support Quality (*significant other)*	-0.60 (-0.71, -0.49)	-0.19 (-0.27, -0.11)
	Support Quality (*family)*	-0.68 (-0.76, -0.60)	-0.30 (-0.37, -0.22)
	Support Quality (*friends)*	-0.69 (-0.81, -0.58)	-0.23 (-0.31, -0.15)
Support Quantity	Support Quality (*total)*	0.64 (0.56, 0.71)	0.38 (0.30, 0.45)
	Support Quality (*significant other)*	0.52 (0.42, 0.62)	0.19 (0.11, 0.27)
	Support Quality (*family)*	0.40 (0.31, 0.50)	0.10 (0.02, 0.19)
	Support Quality (*friends)*	0.81 (0.74, 0.88)	0.54 (0.48, 0.59)

Note. Table showing estimates of genetic and environmental correlation between support and mental health measures. Estimates were obtained through bivariate AE model fitting. Most of the genetic correlations were moderate to high while the non-shared environmental correlations tended to be much lower.

Supplementary Table S9. Proportion of the phenotypic correlation explained by genetic and non-shared environmental overlaps (95% confidence intervals)

		**Proportion of phenotypic correlation explained by genetic overlaps**	**Proportion of the phenotypic correlation explained by non-shared environmental overlaps**	**Phenotypic correlation**
	Support Quantity	0.73 (0.60, 0.84)	0.27 (0.16, 0.40)	0.38 (0.34, 0.41)
	Support Quality (*total)*	0.74 (0.63, 0.84)	0.26 (0.16, 0.37)	0.44 (0.41, 0.47)
Positive Affect	Support Quality (*significant other)*	0.78 (0.64, 0.91)	0.22 (0.09, 0.36)	0.36 (0.32, 0.40)
	Support Quality (*family)*	0.72 (0.60, 0.83)	0.28 (0.17, 0.40)	0.37 (0.33, 0.41)
	Support Quality (*friends)*	0.70 (0.55, 0.84)	0.30 (0.16, 0.45)	0.36 (0.32, 0.39)
	Support Quantity	0.62 (0.36, 0.87)	0.38 (0.13, 0.64)	-0.19 (-0.23, -0.15)
	Support Quality (*total)*	0.74 (0.59, 0.88)	0.26 (0.12, 0.41)	-0.32 (-0.36, -0.28
Negative Affect	Support Quality (*significant other)*	0.77 (0.51, 1.03)	0.23 (-0.03, 0.49)	-0.19 (-0.24, -0.15)
	Support Quality (*family)*	0.72 (0.58, 0.85)	0.28 (0.15, 0.42)	-0.33 (-0.37, -0.29)
	Support Quality (*friends)*	0.72 (0.53, 0.91)	0.28 (0.09, 0.47)	-0.27 (-0.31, -0.23)
	Support Quantity	0.79 (0.65, 0.91)	0.21 (0.09, 0.35)	0.37 (0.33, 0.40)
	Support Quality (*total)*	0.71 (0.62, 0.80)	0.29 (0.20, 0.38)	0.55 (0.52, 0.58)
Subjective Happiness	Support Quality (*significant other)*	0.71 (0.58, 0.82)	0.29 (0.18, 0.42)	0.41 (0.38, 0.45)
	Support Quality (*family)*	0.73 (0.63, 0.82)	0.27 (0.18, 0.37)	0.49 (0.46, 0.52)
	Support Quality (*friends)*	0.71 (0.59, 0.82)	0.29 (0.18, 0.41)	0.45 (0.41, 0.48)
	Support Quantity	0.77 (0.67, 0.86)	0.23 (0.14, 0.33)	0.43 (0.40, 0.47)
Life satisfaction	Support Quality (*total)*	0.68 (0.61, 0.74)	0.32 (0.26, 0.39)	0.68 (0.66, 0.71)
	Support Quality (*significant other)*	0.71 (0.61, 0.80)	0.29 (0.20, 0.39)	0.48 (0.44, 0.51)

	Support Quality (*family)*	0.69 (0.62, 0.76)	0.31 (0.24, 0.38)	0.63 (0.61, 0.66)
	Support Quality (*friends)*	0.64 (0.55, 0.72)	0.36 (0.28, 0.45)	0.59 (0.56, 0.61)
Gratitude	Support Quantity	0.77 (0.64, 0.88)	0.23 (0.12, 0.36)	0.37 (0.33, 0.41)
	Support Quality (*total)*	0.76 (0.67, 0.83)	0.24 (0.17, 0.33)	0.58 (0.55, 0.61)
	Support Quality (*significant other)*	0.75 (0.65, 0.85)	0.25 (0.15, 0.35)	0.45 (0.41, 0.48)
	Support Quality (*family)*	0.78 (0.70, 0.85)	0.22 (0.15, 0.30)	0.57 (0.53, 0.59)
	Support Quality (*friends)*	0.72 (0.60, 0.83)	0.28 (0.17, 0.40)	0.43 (0.39, 0.46)
Meaning in Life	Support Quantity	0.85 (0.72, 0.98)	0.15 (0.02, 0.28)	0.34 (0.30, 0.38)
	Support Quality (*total)*	0.76 (0.68, 0.84)	0.24 (0.16, 0.32)	0.54 (0.51, 0.57)
	Support Quality (*significant other)*	0.80 (0.69, 0.90)	0.20 (0.10, 0.31)	0.44 (0.40, 0.47)
	Support Quality (*family)*	0.75 (0.66, 0.83)	0.25 (0.17, 0.34)	0.48 (0.45, 0.52)
	Support Quality (*friends)*	0.72 (0.60, 0.83)	0.28 (0.17, 0.40)	0.42 (0.38, 0.45)
Autonomy	Support Quantity	0.67 (0.54, 0.79)	0.33 (0.21, 0.46)	0.37 (0.34, 0.41)
	Support Quality (*total)*	0.70 (0.61, 0.78)	0.30 (0.22, 0.39)	0.54 (0.51, 0.57)
	Support Quality (*significant other)*	0.76 (0.64, 0.88)	0.24 (0.12, 0.36)	0.38 (0.34, 0.42)
	Support Quality (*family)*	0.75 (0.66, 0.83)	0.25 (0.17, 0.34)	0.50 (0.47, 0.54)
	Support Quality (*friends)*	0.57 (0.45, 0.68)	0.43 (0.32, 0.55)	0.45 (0.42, 0.49)
Competence	Support Quantity	0.70 (0.57, 0.82)	0.30 (0.18, 0.43)	0.37 (0.33, 0.40)
	Support Quality (*total)*	0.77 (0.68, 0.85)	0.23 (0.15, 0.32)	0.53 (0.50, 0.56)
	Support Quality (*significant other)*	0.81 (0.69, 0.92)	0.19 (0.08, 0.31)	0.40 (0.37, 0.44)
	Support Quality (*family)*	0.79 (0.69, 0.87)	0.21 (0.13, 0.31)	0.48 (0.45, 0.51)

	Support Quality (*friends)*	0.69 (0.57, 0.80)	0.31 (0.20, 0.43)	0.42 (0.38, 0.45)
Relatedness	Support Quantity	0.61 (0.52, 0.69)	0.39 (0.31, 0.48)	0.61 (0.58, 0.64)
	Support Quality (*total)*	0.69 (0.62, 0.76)	0.31 (0.24, 0.38)	0.66 (0.63, 0.68)
	Support Quality (*significant other)*	0.74 (0.64, 0.84)	0.26 (0.16, 0.36)	0.48 (0.44, 0.51)
	Support Quality (*family)*	0.80 (0.71, 0.88)	0.20 (0.12, 0.29)	0.49 (0.46, 0.53)
	Support Quality (*friends)*	0.57 (0.48, 0.65)	0.43 (0.35, 0.52)	0.66 (0.63, 0.68)
Depression	Support Quantity	0.76 (0.61, 0.90)	0.24 (0.10, 0.39)	-0.33 (-0.36, -0.29)
	Support Quality (*total)*	0.69 (0.59, 0.78)	0.31 (0.22, 0.41)	-0.51 (-0.54, -0.48)
	Support Quality (*significant other)*	0.70 (0.56, 0.83)	0.30 (0.17, 0.44)	-0.36 (-0.40, -0.33)
	Support Quality (*family)*	0.69 (0.59, 0.78)	0.31 (0.22, 0.41)	-0.48 (-0.52, -0.45)
	Support Quality (*friends)*	0.66 (0.53, 0.78)	0.34 (0.22, 0.47)	-0.41 (-0.45, -0.38)
Support Quantity	Support Quality (*total)*	0.65 (0.55, 0.73)	0.35 (0.27, 0.45)	0.51 (0.48, 0.54)
	Support Quality (*significant other)*	0.70 (0.56, 0.83)	0.30 (0.17, 0.44)	0.34 (0.31, 0.38)
	Support Quality (*family)*	0.82 (0.66, 0.97)	0.18 (0.03, 0.34)	0.27 (0.23, 0.31)
	Support Quality (*friends)*	0.53 (0.44, 0.61)	0.47 (0.39, 0.56)	0.65 (0.63, 0.68)

Note. Table showing the proportion of the phenotypic correlation between our measures that is accounted for by common genetic or environmental influences. Estimates were obtained through bivariate AE model fitting. Genetic influences explained a larger proportion of the phenotypic correlations between all of the measures of mental health and support

Supplementary Table S10. Skew of measures

**Measure**	**Skew**
Positive Affect	-0.44
Negative Affect	1.20
Subjective Happiness Scale	-0.71
Life Satisfaction Scale	-1.25
Gratitude	-1.04
Meaning in life	-0.87
Basic Psychological Needs: Autonomy	-0.43
Basic Psychological Needs: Competence	-0.38
Basic Psychological Needs: Relatedness	-0.83
Depression	2.20
Support Quantity	-0.53
Support Quality (*total)*	-1.00
Support Quality (*significant other)*	-1.13
Support Quality (*family)*	-1.06
Support Quality (*friends)*	-1.08

Note. Table showing skew of measures. We transformed any measure which had a skew greater than 1 or less than -1.

Supplementary Figure S11: Comparing univariate results (with 95% confidence intervals) of transformed versus untransformed scales

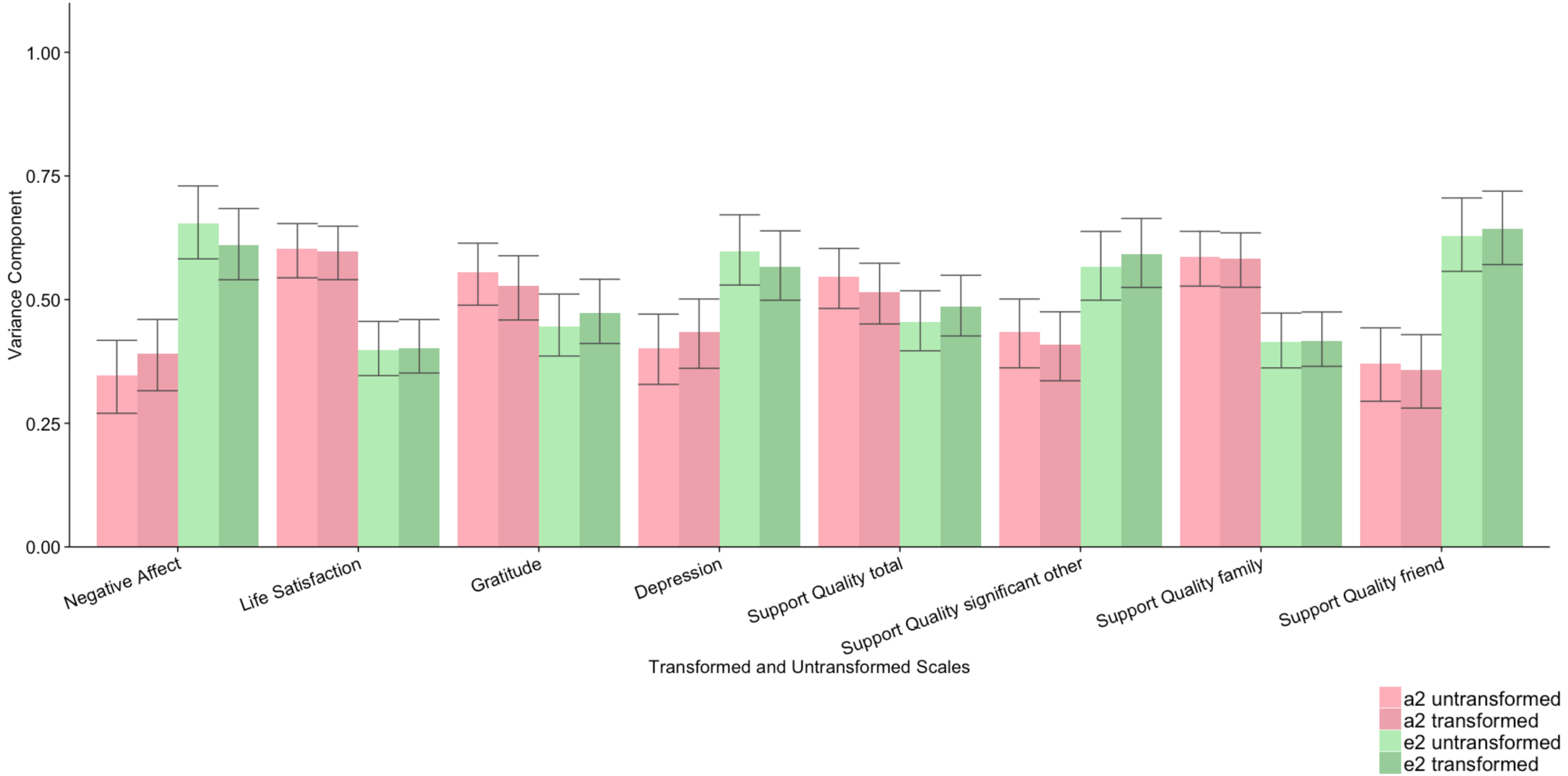

Note. For measures with an absolute skew value over 1, we repeated our analyses on a reflected then log-transformed version of those scales with negative skew and directly log-transformed version of those scales with positive skew. This graph shows the a^2 and e^2 components obtained from our univariate analysis for the untransformed and transformed versions of the scales. Estimates were very similar, all with overlapping confidence intervals.

Supplementary Figure S12. Genetic correlations between transformed and untransformed measures of mental health and support quality (total) with 95% confidence intervals

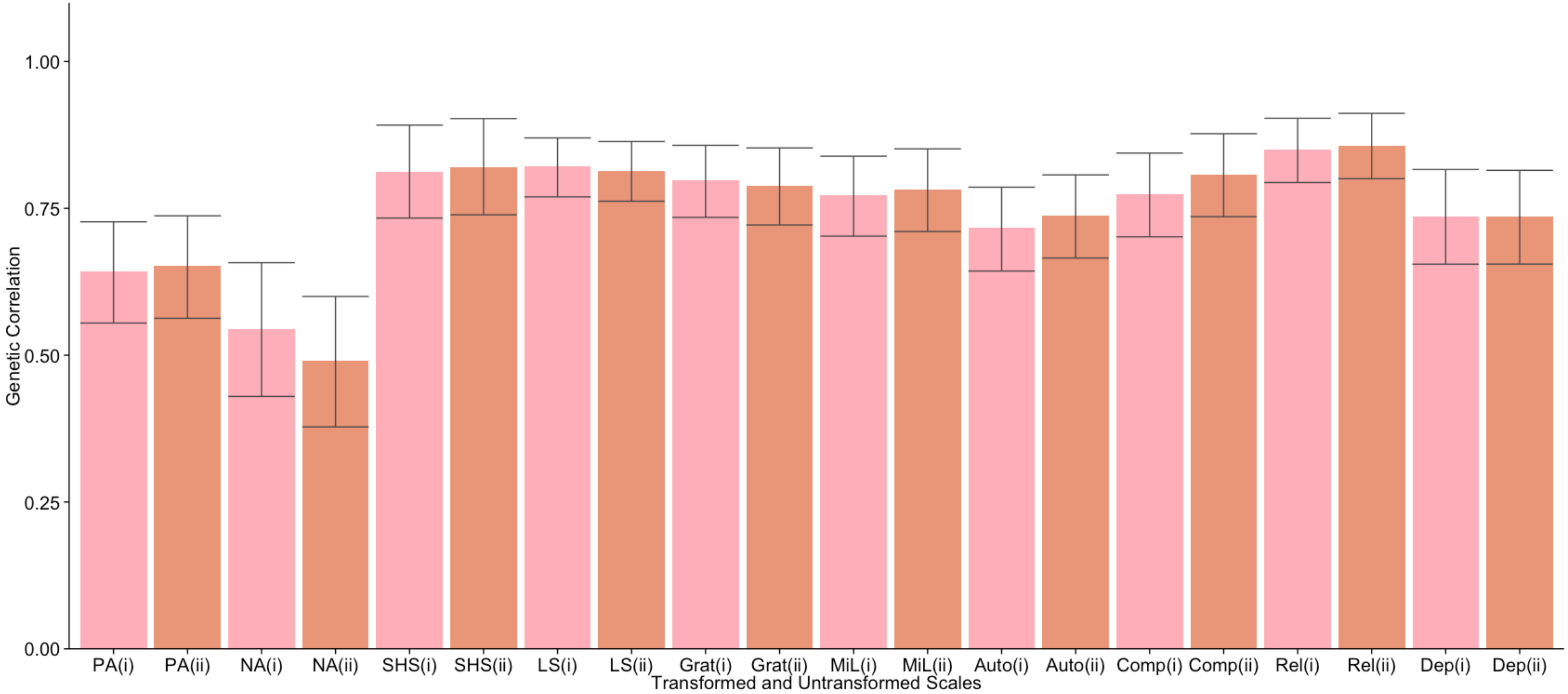

Note. Scales were transformed only when they had an absolute skew value greater than 1. The support quality total score had a negative skew less than -1 meaning that we reflected and then log-transformed this scale. We then obtained the genetic correlation between the untransformed and transformed versions of this scale with untransformed and (where absolute skew was greater than 1) transformed versions of individual mental health measures. For comparative purposes, genetic correlations with negative affect and depression shown here are absolute, as both measures are negatively correlated with support.

Pa(i) = Positive Affect (untransformed) and Support Quality total (untransformed), PA(ii) = Positive Affect (untransformed) and Support Quality total (transformed), NA(i) = Negative Affect (untransformed) and Support Quality total (untransformed), Na(ii) = Negative Affect (transformed) and Support Quality total (transformed), SHS(i) = Subjective Happiness (untransformed) and Support Quality total (untransformed), SHS(ii) = Subjective Happiness (untransformed) and Support Quality total (transformed), LS(i) = Life Satisfaction (untransformed) and Support Quality total (untransformed), LS(ii) = Life Satisfaction (transformed) and Support Quality total (transformed), Grat(i) =

Gratitude (untransformed) and Support Quality total (untransformed), Grat(ii) = Gratitude (transformed) and Support Quality total (transformed), MiL(i) = Meaning in Life (untransformed) and Support Quality total (untransformed), MiL(ii) = Meaning in Life (untransformed) and Support Quality total (transformed), Auto(i) = Autonomy (untransformed) and Support Quality total (untransformed), Auto(ii) = Autonomy (untransformed) and Support Quality total (transformed), Comp(i) = Competence (untransformed) and Support Quality total (untransformed), Comp(ii) = Competence (untransformed) and Support Quality total (transformed), Rel(i) = Relatedness (untransformed) and Support Quality total (untransformed), Rel(ii) = Relatedness (untransformed) and Support Quality total (transformed), Dep(i) = Depression (untransformed) and Support Quality total (untransformed), Dep(ii) = Depression (transformed) and Support Quality total (transformed)

Supplementary Figure S13. Genetic correlations between transformed and untransformed measures of mental health and support quality (significant other subscale) with 95% confidence intervals

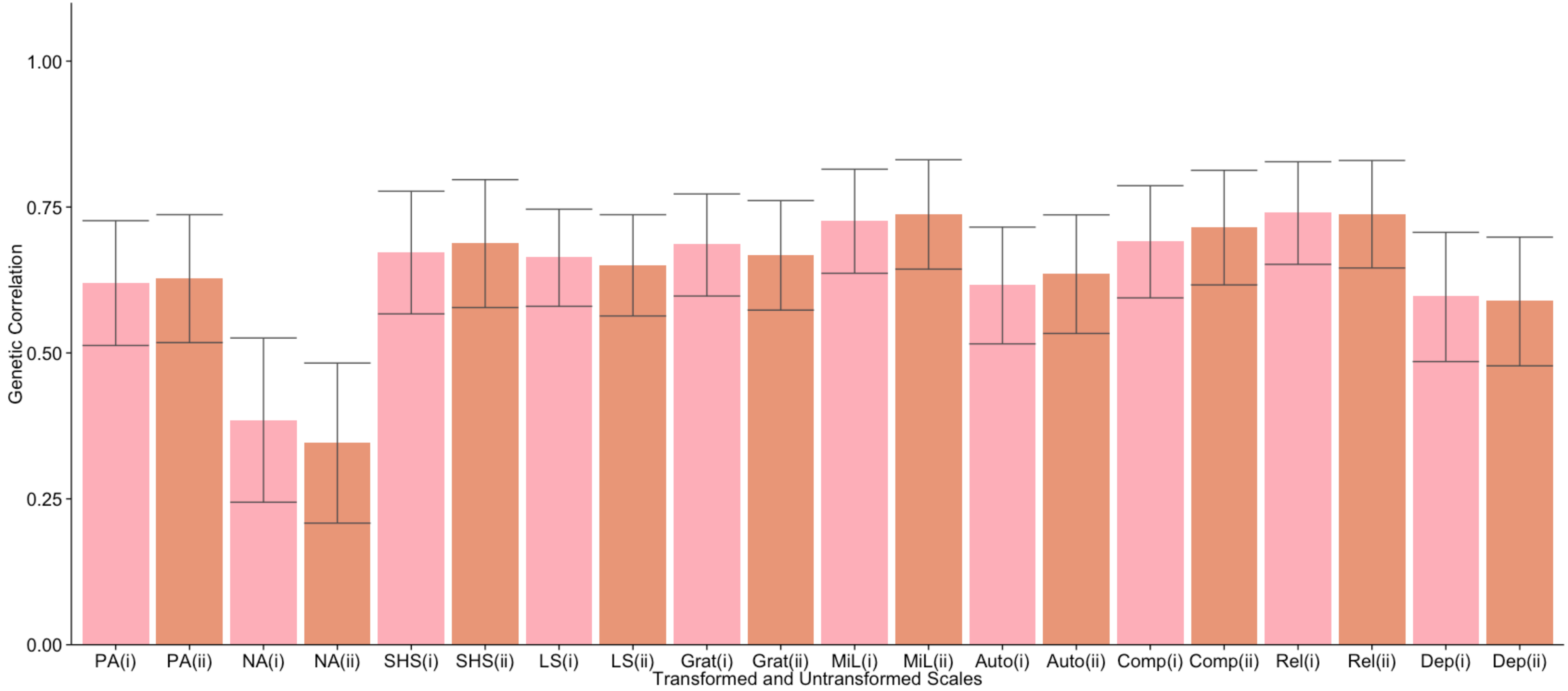

Note. Scales were transformed only when they had an absolute skew value greater than 1. The support quality significant other subscale had a negative skew less than -1 meaning that we reflected and then log-transformed this scale. We then obtained the genetic correlation between the untransformed and transformed versions of this subscale with untransformed and (where absolute skew was greater than 1) transformed versions of individual mental health measures. For comparative purposes, genetic correlations with negative affect and depression shown here are absolute, as both measures are negatively correlated with support.

Pa(i) = Positive Affect (untransformed) and Support Quality significant other (untransformed), PA(ii) = Positive Affect (untransformed) and Support Quality significant other (transformed), NA(i) = Negative Affect (untransformed) and Support Quality significant other (untransformed), Na(ii) = Negative Affect (transformed) and Support Quality significant other (transformed), SHS(i) = Subjective Happiness (untransformed) and Support Quality significant other (untransformed), SHS(ii) = Subjective Happiness (untransformed) and Support Quality significant other (transformed), LS(i) = Life Satisfaction (untransformed) and Support Quality significant other (untransformed), LS(ii) =

Life Satisfaction (transformed) and Support Quality significant other (transformed), Grat(i) = Gratitude (untransformed) and Support Quality significant other (untransformed), Grat(ii) = Gratitude (transformed) and Support Quality significant other (transformed), MiL(i) = Meaning in Life (untransformed) and Support Quality significant other (untransformed), MiL(ii) = Meaning in Life (untransformed) and Support Quality significant other (transformed), Auto(i) = Autonomy (untransformed) and Support Quality significant other (untransformed), Auto(ii) = Autonomy (untransformed) and Support Quality significant other (transformed), Comp(i) = Competence (untransformed) and Support Quality significant other (untransformed), Comp(ii) = Competence (untransformed) and Support Quality significant other (transformed), Rel(i) = Relatedness (untransformed) and Support Quality significant other (untransformed), Rel(ii) = Relatedness (untransformed) and Support Quality significant other (transformed), Dep(i) = Depression (untransformed) and Support Quality significant other (untransformed), Dep(ii) = Depression (transformed) and Support Quality significant other (transformed)

Supplementary Figure S14. Genetic correlations between transformed and untransformed measures of mental health and support quality (family subscale) with 95% confidence intervals

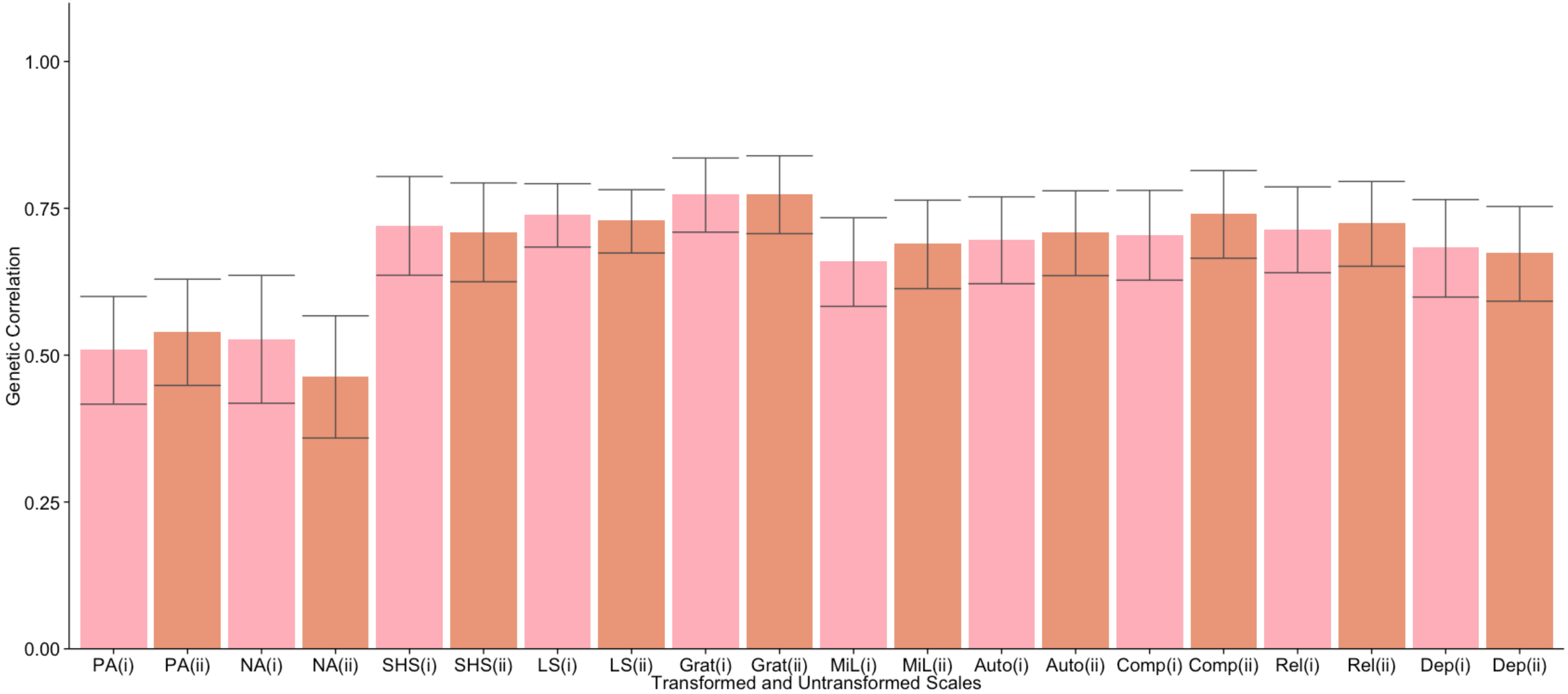

Note. Scales were transformed only when they had an absolute skew value greater than 1. The support quality family subscale had a negative skew less than -1 meaning that we reflected and then log-transformed this scale. We then obtained the genetic correlation between the untransformed and transformed versions of this subscale with untransformed and (where absolute skew was greater than 1) transformed versions of individual mental health measures. For comparative purposes, genetic correlations with negative affect and depression shown here are absolute, as both measures are negatively correlated with support.

Pa(i) = Positive Affect (untransformed) and Support Quality family (untransformed), PA(ii) = Positive Affect (untransformed) and Support Quality family (transformed), NA(i) = Negative Affect (untransformed) and Support Quality family (untransformed), Na(ii) = Negative Affect (transformed) and Support Quality family (transformed), SHS(i) = Subjective Happiness (untransformed) and Support Quality family (untransformed), SHS(ii) = Subjective Happiness (untransformed) and Support Quality family (transformed), LS(i) = Life Satisfaction (untransformed) and Support Quality family (untransformed), LS(ii) = Life Satisfaction (transformed) and Support Quality family

(transformed), Grat(i) = Gratitude (untransformed) and Support Quality family (untransformed), Grat(ii) = Gratitude (transformed) and Support Quality family (transformed), MiL(i) = Meaning in Life (untransformed) and Support Quality family (untransformed), MiL(ii) = Meaning in Life (untransformed) and Support Quality family (transformed), Auto(i) = Autonomy (untransformed) and Support Quality family (untransformed), Auto(ii) = Autonomy (untransformed) and Support Quality family (transformed), Comp(i) = Competence (untransformed) and Support Quality family (untransformed), Comp(ii) = Competence (untransformed) and Support Quality family (transformed), Rel(i) = Relatedness (untransformed) and Support Quality family (untransformed), Rel(ii) = Relatedness (untransformed) and Support Quality family (transformed), Dep(i) = Depression (untransformed) and Support Quality family (untransformed), Dep(ii) = Depression (transformed) and Support Quality family (transformed)

Supplementary Figure S15. Genetic correlations between transformed and untransformed measures of mental health and support quality (friends subscale) with 95% confidence intervals

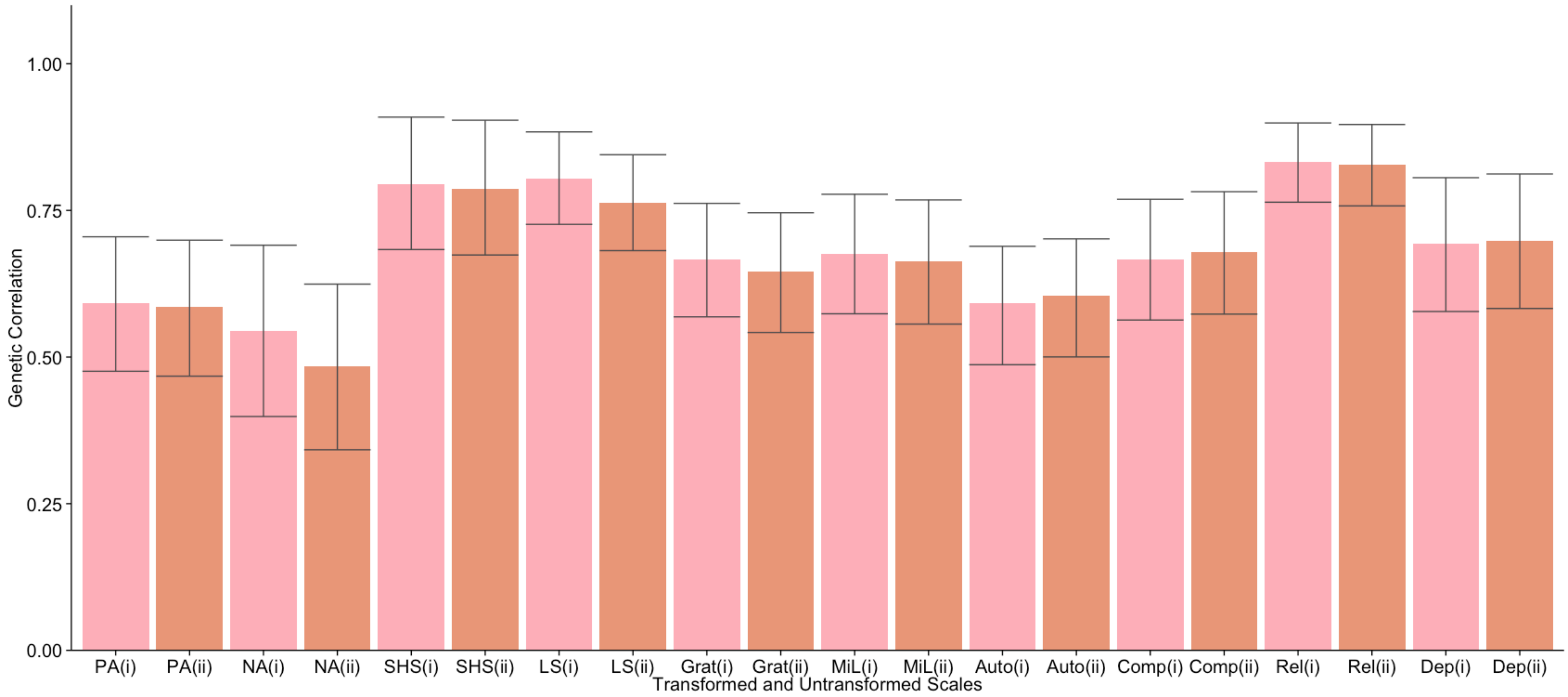

Note. Scales were transformed only when they had an absolute skew value greater than 1. The support quality friends subscale had a negative skew less than -1 meaning that we reflected and then log-transformed this scale. We then obtained the genetic correlation between the untransformed and transformed versions of this subscale with untransformed and (where absolute skew was greater than 1) transformed versions of individual mental health measures. For comparative purposes, genetic correlations with negative affect and depression shown here are absolute, as both measures are negatively correlated with support.

Pa(i) = Positive Affect (untransformed) and Support Quality friends (untransformed), PA(ii) = Positive Affect (untransformed) and Support Quality friends (transformed), NA(i) = Negative Affect (untransformed) and Support Quality friends (untransformed), Na(ii) = Negative Affect (transformed) and Support Quality friends (transformed), SHS(i) = Subjective Happiness (untransformed) and Support Quality friends (untransformed), SHS(ii) = Subjective Happiness (untransformed) and Support Quality friends (transformed), LS(i) = Life Satisfaction (untransformed) and Support Quality friends (untransformed), LS(ii) = Life Satisfaction (transformed) and Support Quality friends

(transformed), Grat(i) = Gratitude (untransformed) and Support Quality friends (untransformed), Grat(ii) = Gratitude (transformed) and Support Quality friends (transformed), MiL(i) = Meaning in Life (untransformed) and Support Quality friends (untransformed), MiL(ii) = Meaning in Life (untransformed) and Support Quality friends (transformed), Auto(i) = Autonomy (untransformed) and Support Quality friends (untransformed), Auto(ii) = Autonomy (untransformed) and Support Quality friends (transformed), Comp(i) = Competence (untransformed) and Support Quality friends (untransformed), Comp(ii) = Competence (untransformed) and Support Quality friends (transformed), Rel(i) = Relatedness (untransformed) and Support Quality friends (untransformed), Rel(ii) = Relatedness (untransformed) and Support Quality friends (transformed), Dep(i) = Depression (untransformed) and Support Quality friends (untransformed), Dep(ii) = Depression (transformed) and Support Quality friends (transformed)

Supplementary Figure S16. Genetic correlations between transformed and untransformed measures of mental health and support quantity with 95% confidence intervals

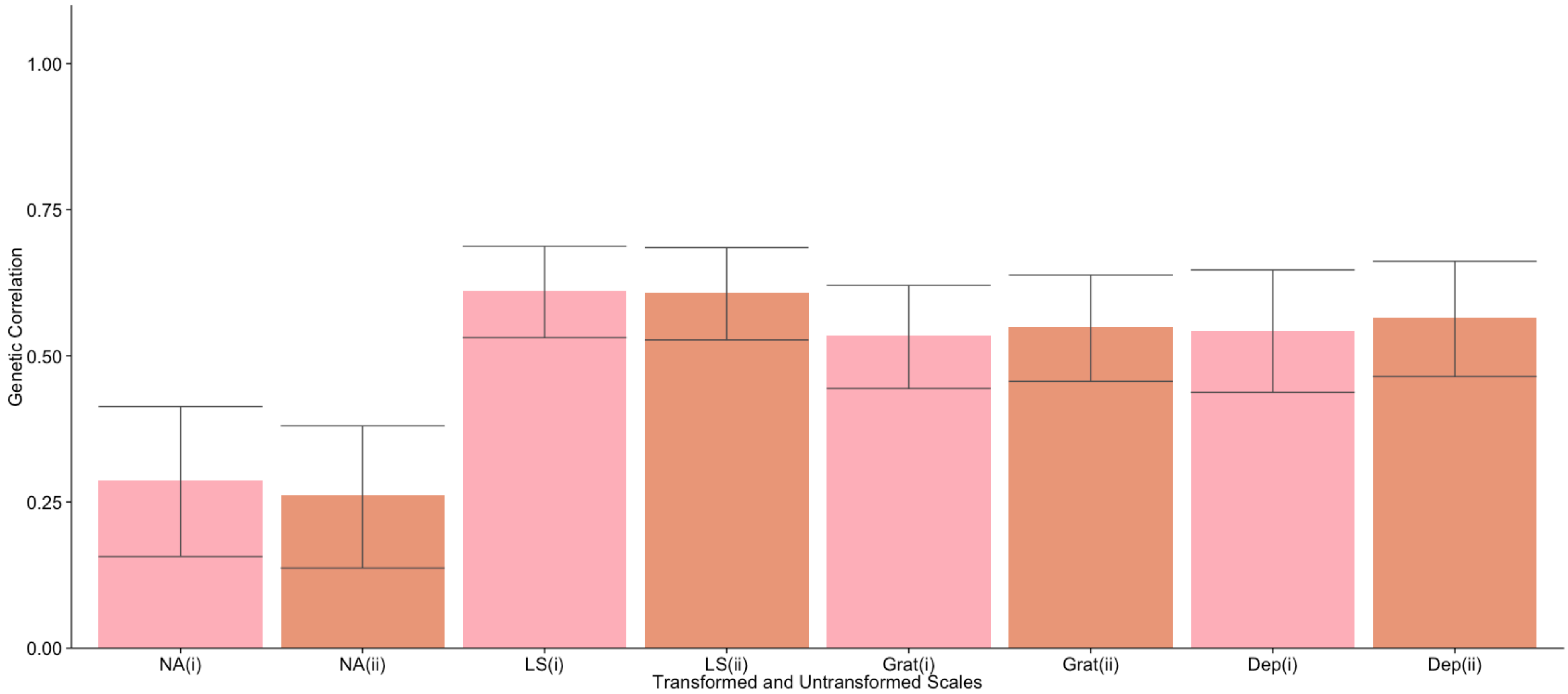

Note. Support quantity had a skew of between 0 and -1 so was not transformed. We obtained the genetic correlation between this untransformed scale with untransformed and (where absolute skew was greater than 1) transformed versions of individual mental health measures. For comparative purposes, genetic correlations with negative affect and depression shown here are absolute, as both measures are negatively correlated with support.

NA(i) = Negative Affect (untransformed) and Support Quantity (untransformed), Na(ii) = Negative Affect (transformed) and Support Quantity (transformed), LS(i) = Life Satisfaction (untransformed) and Support Quantity (untransformed), LS(ii) = Life Satisfaction (transformed) and Support Quantity (transformed), Grat(i) = Gratitude (untransformed) and Support Quantity (untransformed), Grat(ii) = Gratitude (transformed) and Support Quantity (transformed), Dep(i) = Depression (untransformed) and Support Quantity (untransformed), Dep(ii) = Depression (transformed) and Support Quantity (transformed)

Supplementary Figure S17. Environmental correlations between transformed and untransformed measures of mental health and support quality (total) with 95% confidence intervals

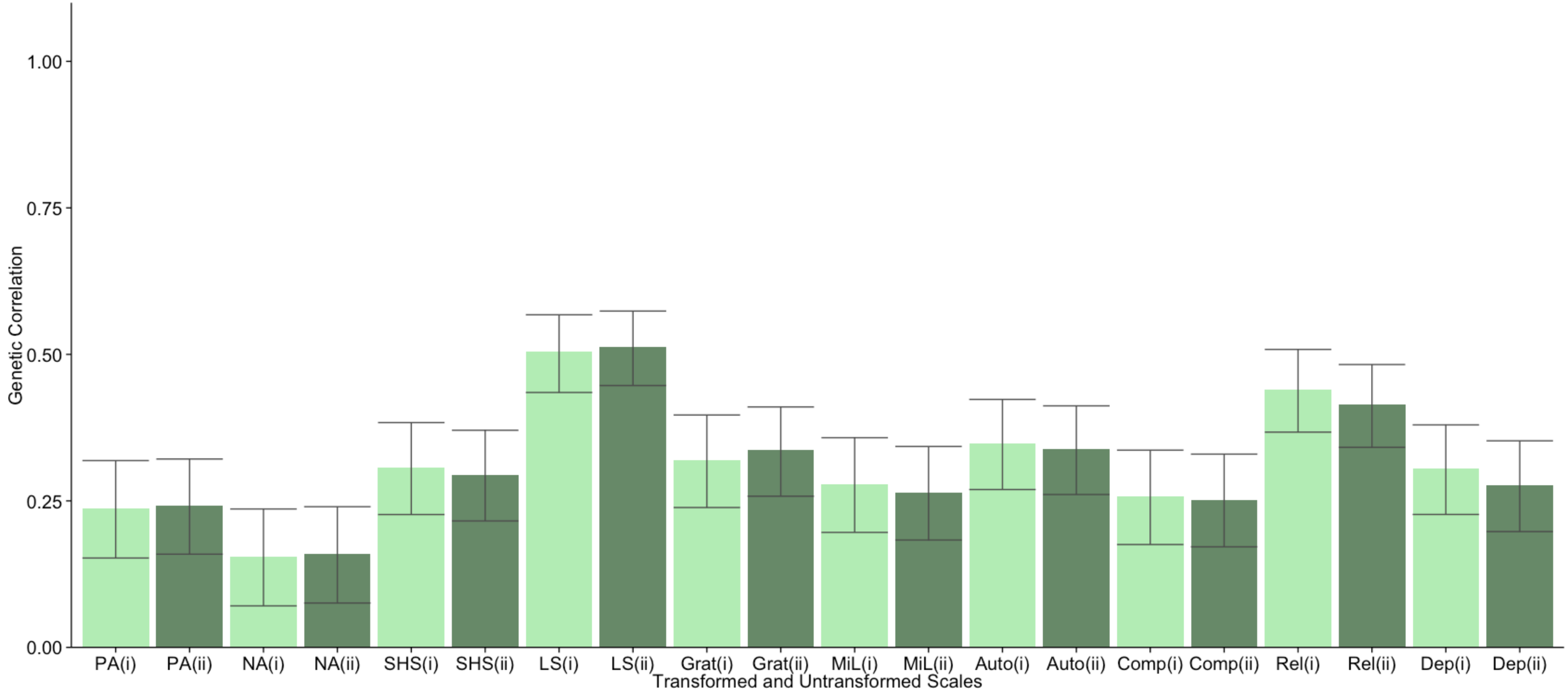

Note. Scales were transformed only when they had an absolute skew value greater than 1. The support quality total score had a negative skew less than -1 meaning that we reflected and then log-transformed this scale. We then obtained the non-shared environmental correlation between the untransformed and transformed versions of this scale with untransformed and (where absolute skew was greater than 1) transformed versions of individual mental health measures. For comparative purposes, non-shared environmental correlations with negative affect and depression shown here are absolute, as both measures are negatively correlated with support.

Pa(i) = Positive Affect (untransformed) and Support Quality total (untransformed), PA(ii) = Positive Affect (untransformed) and Support Quality total (transformed), NA(i) = Negative Affect (untransformed) and Support Quality total (untransformed), Na(ii) = Negative Affect (transformed) and Support Quality total (transformed), SHS(i) = Subjective Happiness (untransformed) and Support Quality total (untransformed), SHS(ii) = Subjective Happiness (untransformed) and Support Quality total (transformed),

LS(i) = Life Satisfaction (untransformed) and Support Quality total (untransformed), LS(ii) = Life Satisfaction (transformed) and Support Quality total (transformed), Grat(i) = Gratitude (untransformed) and Support Quality total (untransformed), Grat(ii) = Gratitude (transformed) and Support Quality total (transformed), MiL(i) = Meaning in Life (untransformed) and Support Quality total (untransformed), MiL(ii) = Meaning in Life (untransformed) and Support Quality total (transformed), Auto(i) = Autonomy (untransformed) and Support Quality total (untransformed), Auto(ii) = Autonomy (untransformed) and Support Quality total (transformed), Comp(i) = Competence (untransformed) and Support Quality total (untransformed), Comp(ii) = Competence (untransformed) and Support Quality total (transformed), Rel(i) = Relatedness (untransformed) and Support Quality total (untransformed), Rel(ii) = Relatedness (untransformed) and Support Quality total (transformed), Dep(i) = Depression (untransformed) and Support Quality total (untransformed), Dep(ii) = Depression (transformed) and Support Quality total (transformed)

Supplementary Figure S18. Environmental correlations between transformed and untransformed measures of mental health and support quantity (significant other) with 95% confidence intervals

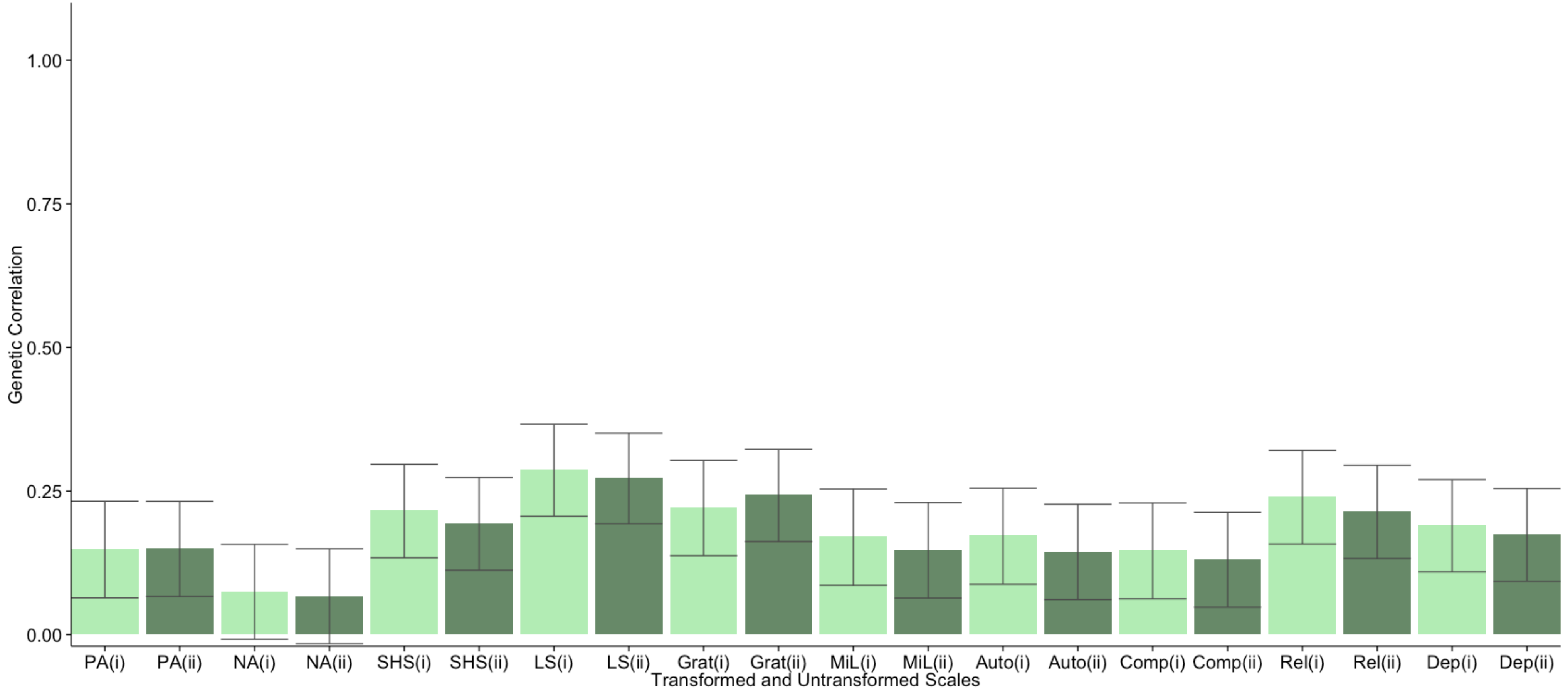

Note. Scales were transformed only when they had an absolute skew value greater than 1. The support quality significant other subscale had a negative skew less than -1 meaning that we reflected and then log-transformed this subscale. We then obtained the non-shared environmental correlation between the untransformed and transformed versions of this subscale with untransformed and (where absolute skew was greater than 1) transformed versions of individual mental health measures. For comparative purposes, non-shared environmental correlations with negative affect and depression shown here are absolute, as both measures are negatively correlated with support. Confidence interval for negative affect shows that they cross 0.

Pa(i) = Positive Affect (untransformed) and Support Quality significant other (untransformed), PA(ii) = Positive Affect (untransformed) and Support Quality significant other (transformed), NA(i) = Negative Affect (untransformed) and Support Quality significant other (untransformed), Na(ii) = Negative Affect (transformed) and Support Quality

significant other (transformed), SHS(i) = Subjective Happiness (untransformed) and Support Quality significant other (untransformed), SHS(ii) = Subjective Happiness (untransformed) and Support Quality significant other (transformed), LS(i) = Life Satisfaction (untransformed) and Support Quality significant other (untransformed), LS(ii) = Life Satisfaction (transformed) and Support Quality significant other (transformed), Grat(i) = Gratitude (untransformed) and Support Quality significant other (untransformed), Grat(ii) = Gratitude (transformed) and Support Quality significant other (transformed), MiL(i) = Meaning in Life (untransformed) and Support Quality significant other (untransformed), MiL(ii) = Meaning in Life (untransformed) and Support Quality significant other (transformed), Auto(i) = Autonomy (untransformed) and Support Quality significant other (untransformed), Auto(ii) = Autonomy (untransformed) and Support Quality significant other (transformed), Comp(i) = Competence (untransformed) and Support Quality significant other (untransformed), Comp(ii) = Competence (untransformed) and Support Quality significant other (transformed), Rel(i) = Relatedness (untransformed) and Support Quality significant other (untransformed), Rel(ii) = Relatedness (untransformed) and Support Quality significant other (transformed), Dep(i) = Depression (untransformed) and Support Quality significant other (untransformed), Dep(ii) = Depression (transformed) and Support Quality significant other (transformed)

Supplementary Figure S19. Environmental correlations between transformed and untransformed measures of mental health and support quantity (family) with 95% confidence intervals

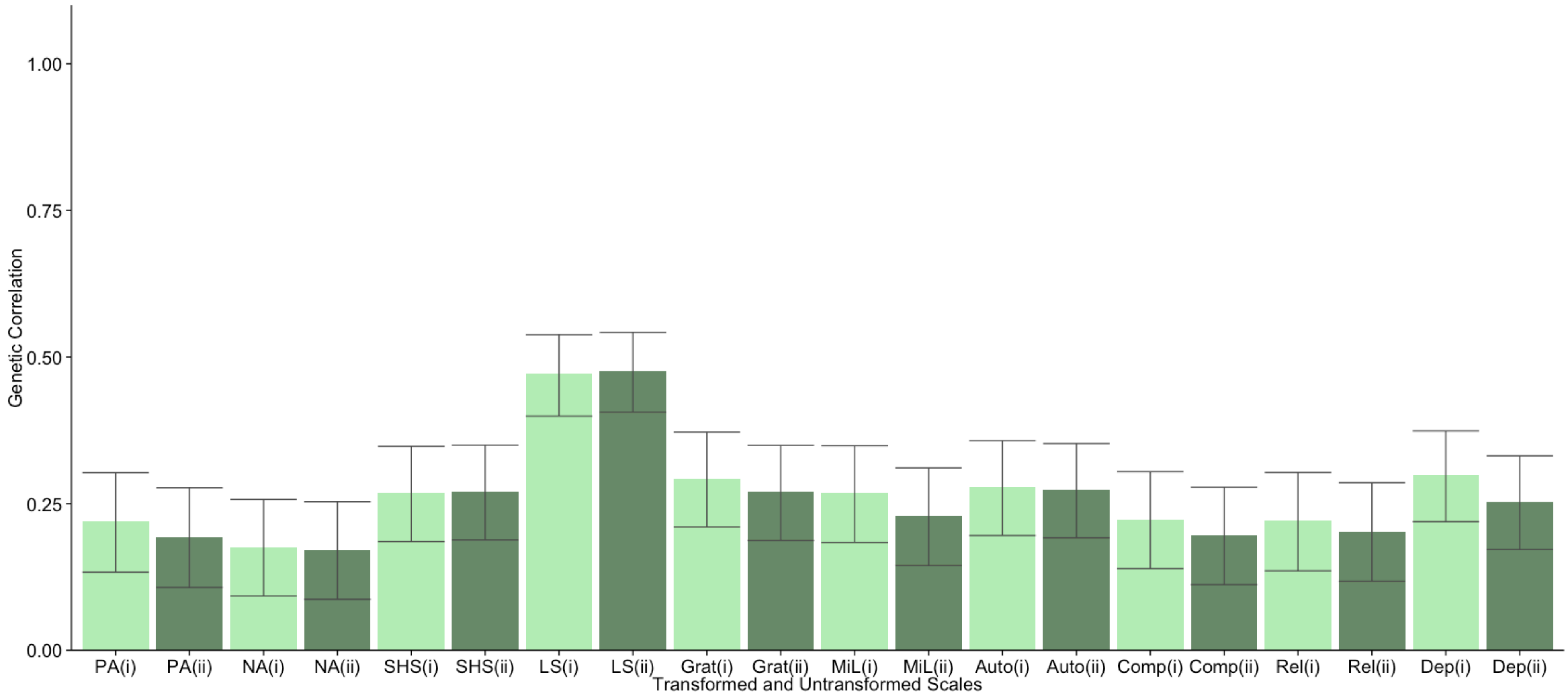

Note. Scales were transformed only when they had an absolute skew value greater than 1. The support quality family subscale had a negative skew less than -1 meaning that we reflected and then log-transformed this subscale. We then obtained the non-shared environmental correlation between the untransformed and transformed versions of this subscale with untransformed and (where absolute skew was greater than 1) transformed versions of individual mental health measures. For comparative purposes, non-shared environmental correlations with negative affect and depression shown here are absolute, as both measures are negatively correlated with support.

Pa(i) = Positive Affect (untransformed) and Support Quality family (untransformed), PA(ii) = Positive Affect (untransformed) and Support Quality family (transformed), NA(i) = Negative Affect (untransformed) and Support Quality family (untransformed), Na(ii) = Negative Affect (transformed) and Support Quality family (transformed), SHS(i) = Subjective Happiness (untransformed) and Support Quality family (untransformed), SHS(ii) = Subjective Happiness (untransformed) and Support Quality family (transformed), LS(i) = Life Satisfaction (untransformed) and Support Quality family (untransformed), LS(ii) = Life Satisfaction (transformed) and Support Quality family

(transformed), Grat(i) = Gratitude (untransformed) and Support Quality family (untransformed), Grat(ii) = Gratitude (transformed) and Support Quality family (transformed), MiL(i) = Meaning in Life (untransformed) and Support Quality family (untransformed), MiL(ii) = Meaning in Life (untransformed) and Support Quality family (transformed), Auto(i) = Autonomy (untransformed) and Support Quality family (untransformed), Auto(ii) = Autonomy (untransformed) and Support Quality family (transformed), Comp(i) = Competence (untransformed) and Support Quality family (untransformed), Comp(ii) = Competence (untransformed) and Support Quality family (transformed), Rel(i) = Relatedness (untransformed) and Support Quality family (untransformed), Rel(ii) = Relatedness (untransformed) and Support Quality family (transformed), Dep(i) = Depression (untransformed) and Support Quality family (untransformed), Dep(ii) = Depression (transformed) and Support Quality family (transformed)

Supplementary Figure S20. Environmental correlations between transformed and untransformed measures of mental health and support quantity (friends) with 95% confidence intervals

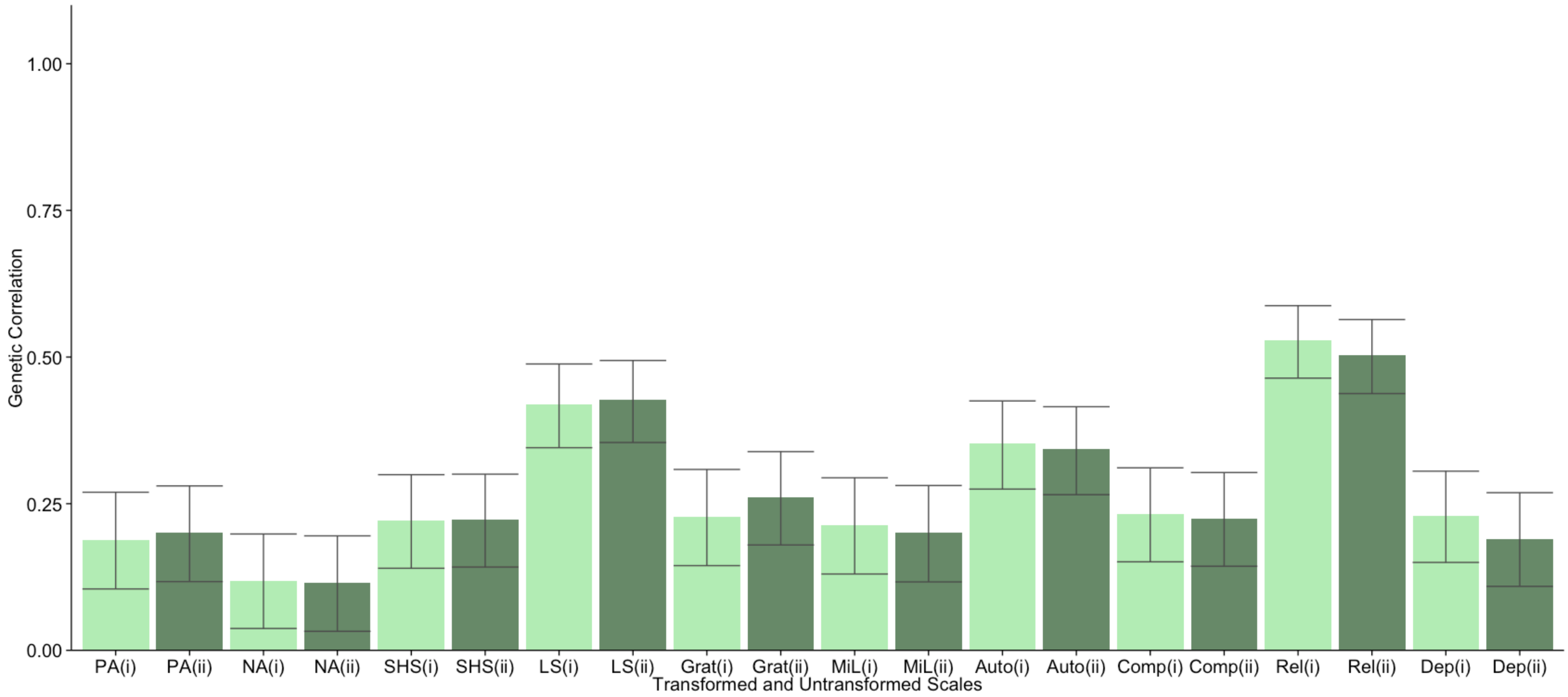

Note. Scales were transformed only when they had an absolute skew value greater than 1. The support quality friends subscale had a negative skew less than -1 meaning that we reflected and then log-transformed this subscale. We then obtained the non-shared environmental correlation between the untransformed and transformed versions of this subscale with untransformed and (where absolute skew was greater than 1) transformed versions of individual mental health measures. For comparative purposes, non-shared environmental correlations with negative affect and depression shown here are absolute, as both measures are negatively correlated with support.

Pa(i) = Positive Affect (untransformed) and Support Quality friends (untransformed), PA(ii) = Positive Affect (untransformed) and Support Quality friends (transformed), NA(i) = Negative Affect (untransformed) and Support Quality friends (untransformed), Na(ii) = Negative Affect (transformed) and Support Quality friends (transformed), SHS(i) = Subjective Happiness (untransformed) and Support Quality friends (untransformed), SHS(ii) = Subjective Happiness (untransformed) and Support Quality friends (transformed), LS(i) = Life Satisfaction (untransformed) and Support Quality friends (untransformed), LS(ii) = Life Satisfaction (transformed) and Support Quality friends

(transformed), Grat(i) = Gratitude (untransformed) and Support Quality friends (untransformed), Grat(ii) = Gratitude (transformed) and Support Quality friends (transformed), MiL(i) = Meaning in Life (untransformed) and Support Quality friends (untransformed), MiL(ii) = Meaning in Life (untransformed) and Support Quality friends (transformed), Auto(i) = Autonomy (untransformed) and Support Quality friends (untransformed), Auto(ii) = Autonomy (untransformed) and Support Quality friends (transformed), Comp(i) = Competence (untransformed) and Support Quality friends (untransformed), Comp(ii) = Competence (untransformed) and Support Quality friends (transformed), Rel(i) = Relatedness (untransformed) and Support Quality friends (untransformed), Rel(ii) = Relatedness (untransformed) and Support Quality friends (transformed), Dep(i) = Depression (untransformed) and Support Quality friends (untransformed), Dep(ii) = Depression (transformed) and Support Quality friends (transformed)

Supplementary Figure S21. Environmental correlations between transformed and untransformed measures of mental health and support quantity with 95% confidence intervals

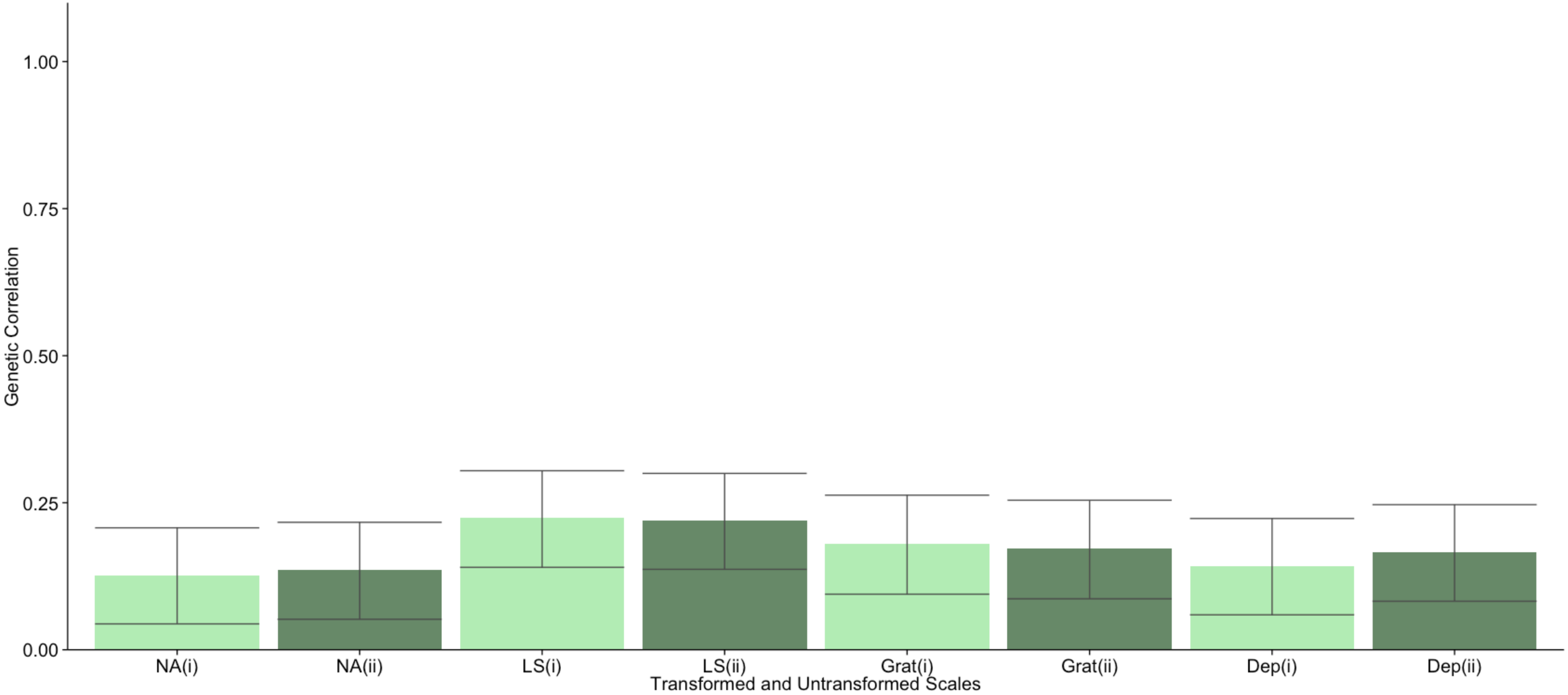

Note. Support quantity had a skew of between 0 and -1 so was not transformed. We obtained the non-shared environmental correlation between this untransformed scale with untransformed and (where absolute skew was greater than 1) transformed versions of individual mental health measures. For comparative purposes, non-shared environmental correlations with negative affect and depression shown here are absolute, as both measures are negatively correlated with support.

NA(i) = Negative Affect (untransformed) and Support Quantity (untransformed), Na(ii) = Negative Affect (transformed) and Support Quantity (transformed), LS(i) = Life Satisfaction (untransformed) and Support Quantity (untransformed), LS(ii) = Life Satisfaction (transformed) and Support Quantity (transformed), Grat(i) = Gratitude (untransformed) and Support Quantity (untransformed), Grat(ii) = Gratitude (transformed) and Support Quantity (transformed), Dep(i) = Depression (untransformed) and Support Quantity (untransformed), Dep(ii) = Depression (transformed) and Support Quantity (transformed)

Supplementary Figure S22: Illustration of the basic twin model for one trait of one twin (within a twin pair)

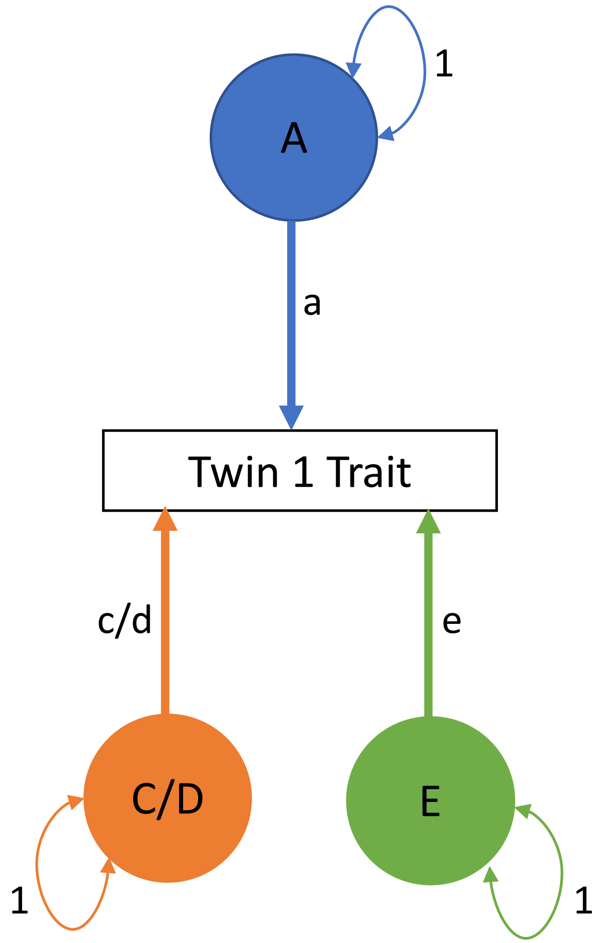

Additive genetic influence, A:

$a*1*a = a^2$

Non-additive genetic influence, D:

$d*1*d = d^2$

Shared environmental influence, C:

$c*1*c = c^2$

Non-shared environmental influence, E:

$e*1*e = e^2$

Note. This is the basic twin model path diagram for a trait of one twin (within a twin pair). a, d/c and e are path coefficients. The variance of a variable is the covariance of the variable with itself so the expected additive genetic (A), non-additive genetic (D)/shared environmental (C), and non-shared environmental (E) influences are the individual paths from the variable to itself (following Wright's rules of path tracing). a^2, d^2/c^2 and e^2 are the indices of the relative contribution of A, D/C and E respectively on the phenotypic variance. The phenotypic variance is equal to the sum of all paths.

Supplementary Figure S23. A path diagram of the basic twin model

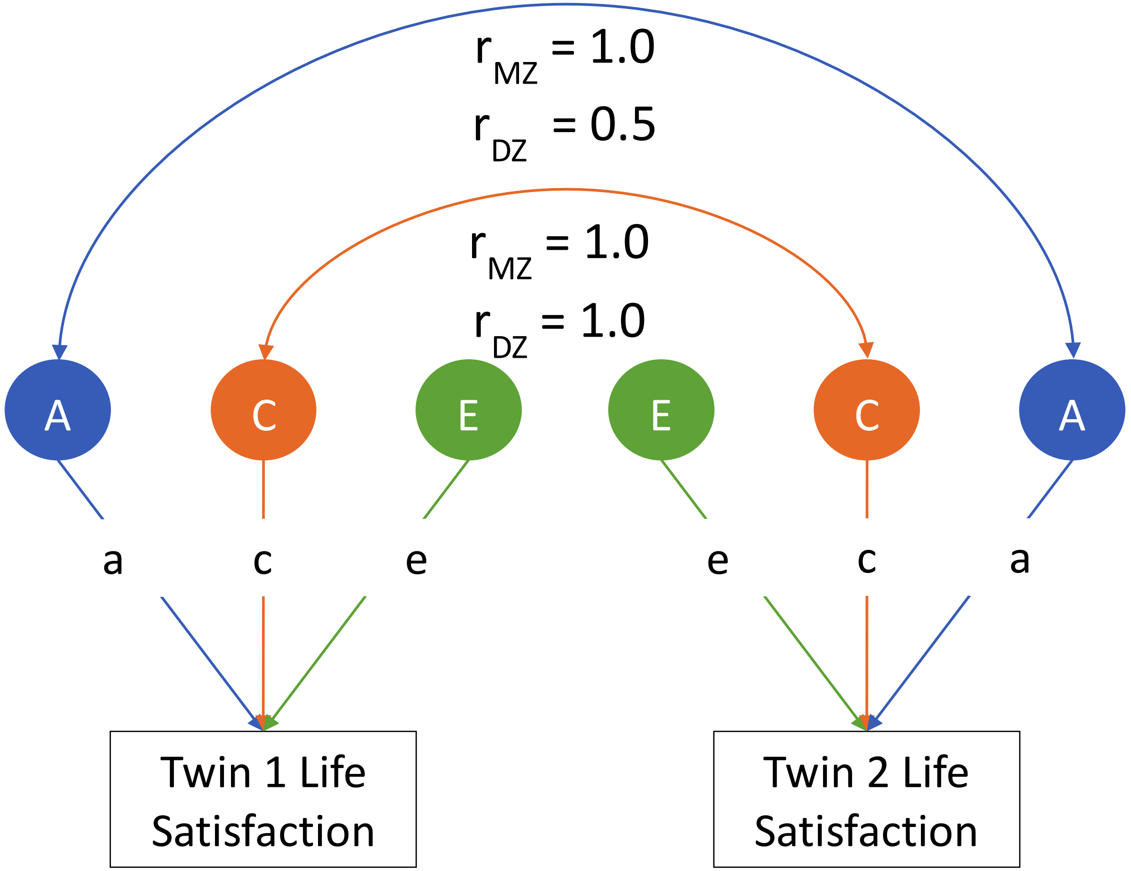

Falconer's equations:

$$A + C = r_{MZ}$$

$$0.5*A + C = r_{DZ}$$

$$A + C + E = 1$$

Falconer's equations can be rewritten as:

$$A = 2(r_{MZ} - r_{DZ})$$

$$C = r_{MZ} – A$$

$$E = 1- (A + C)$$

Note. This figure shows the genetic and environmental relationships within twins for one trait and forms the basis of all twin analyses. The total observed phenotypic variance of a trait, shown by the rectangular boxes, is the sum of additive genetic (A), shared environmental (C), and non-shared environmental (E) influences, shown as latent factors in the circles. The path coefficients of these latent variables are represented by a, c and e respectively. We know that MZ twins are 100% genetically identical while DZ twins are on average 50% genetically identical. We also assume that both reared together MZ and DZ twins share 100% of their shared environment. Using this information of genetic and environmental similarity, we can predict concordances of a trait between MZ and DZ twin pairs and compare this to actual observed concordances to enable us to decompose the phenotypic variance of a trait using simultaneous equations (Falconer's equations). When non-additive genetic (D) are used instead of C, rMZ = 1.0 and rDZ=0.25.

Supplementary Figure S24: Illustration of the Cholesky Decomposition

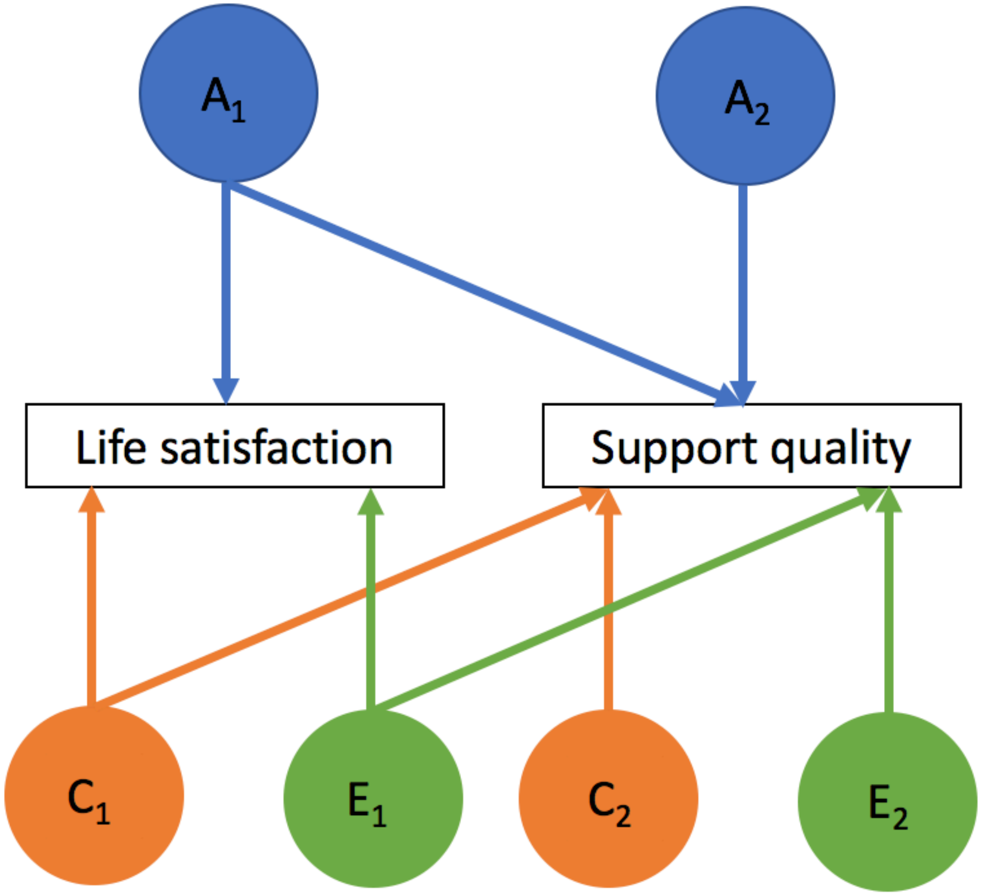

Note. A bivariate Cholesky decomposition allows us to decompose the covariance between two traits, indicating the degree of genetic and environmental overlap between our measures. The first genetic factor (A1) represents genetic influences on life satisfaction. The extent to which these same genes also influence support quality is estimated (displayed by the diagonal pathway). The second genetic factor (A2) represents the genetic influence on support quality, which is independent of genetic influences shared with life satisfaction. The same decomposition is done for shared and non-shared environmental influences.

Supplementary Figure S25: Illustration of the Correlated Factors Solution

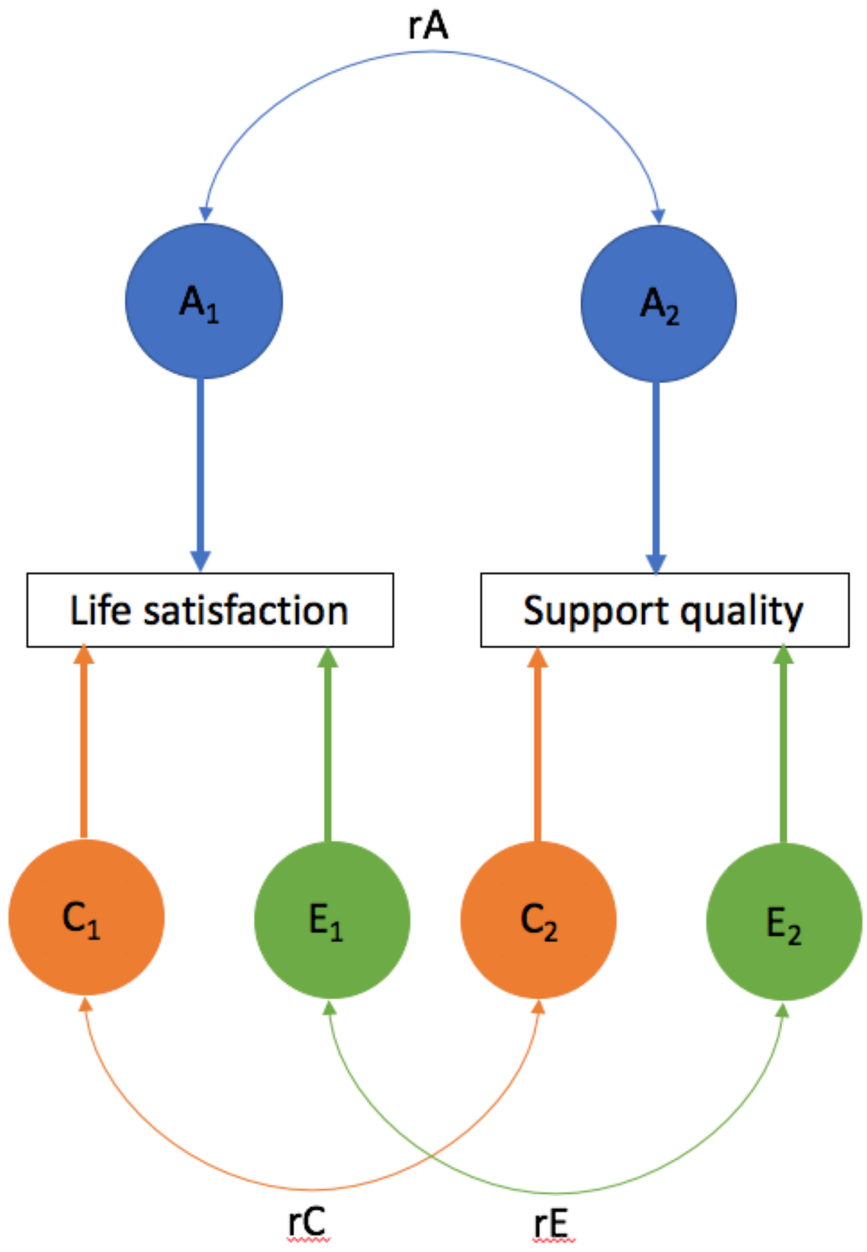

Note. We can convert the results obtained from a bivariate Cholesky decomposition into the mathematically equivalent correlated factors solution, which does not impose an order on the included variables and allows us to estimate genetic and environmental correlations between our measures. We can also calculate the proportion of the phenotypic correlation between our measures of mental health and support that is accounted for by overlapping genetic or environmental influences.
